# Association Between ABO Blood Groups and *Helicobacter pylori* Infection: A Meta-Analysis

**DOI:** 10.1038/s41598-018-36006-x

**Published:** 2018-12-04

**Authors:** Zakaria Chakrani, Karen Robinson, Bineyam Taye

**Affiliations:** 1 Department of Biology, Colgate University, 13 Oak Dr, Hamilton, 13346 NY USA; 2 NIHR Nottingham Biomedical Research Centre, Nottingham University Hospitals NHS Trust and The University of Nottingham, Nottingham, UK; 3 Nottingham Digestive Diseases Centre, The University of Nottingham, Nottingham, UK

## Abstract

There is no consensus among the existing literature on the relationship between ABO blood groups and risk of *Helicobacter pylori* infection. However, histo-blood group carbohydrates are proposed to influence the risk of acquiring this pathogen via effects on adhesion to the gastric mucosa. The objective of this meta-analysis was to evaluate the association between ABO blood groups and *H*. *pylori* infection. All relevant epidemiological studies published in English (up to October 2017) was retrieved through an extensive systematic literature search of MEDLINE/PubMed databases. Pooled estimates of effects were obtained through the use of fixed and random effects meta-analyses. Individuals with O blood group were more likely to be infected with *H*. *pylori* (pooled odds ratio (OR) 1.163; 95% confidence interval (CI) 1.074–1.259; P < 0.001). While individuals with B and AB blood group were less likely to be infected with *H*. *pylori* (OR 0.831; 95% CI 0.738–0.935; P = 0.002 and OR 0.709; 95% CI 0.605–0.832; P < 0.001, respectively). The results from this meta-analysis of observational studies suggest an estimated 16.3% increased odds of *H*. *pylori* infection amongst individuals with the O blood group. If this observed association is causal, a better understanding of the underlying mechanisms could provide indications to potential prevention strategies for *H*. *pylori* infection.

## Introduction

*Helicobacter pylori* is a Gram-negative spiral-shaped pathogenic bacterium which inhabits the human gastric mucosa. The bacterium is present in approximately half of the world’s population^1,2^, but it causes symptomatic disease (peptic ulcer disease and gastric malignancy) in only 10–15% of those infected^3,4^. In 1994, *H*. *pylori* was categorized as a class I carcinogen by the International Agency for Research on Cancer (IARC), a division of the World Health Organization (WHO) and considered to be the major risk factor of gastric cancer^5^. Understanding who is likely to become infected and develop disease is extremely important, especially with increasingly common antibiotic resistant strains and the lack of an effective vaccine^6^.

The mechanisms by which *H*. *pylori* is usually acquired and its route of transmission remain unclear, however close human contact is required. Previous epidemiological studies showed that household hygiene practices and socioeconomic status (as defined by occupation, family income level, and living conditions) are important risk factors for *H*. *pylori* infection^7,8^. These factors are thought to partially explain why rates of *H*. *pylori* infection vary between populations. Furthermore, a recent study showed a significant higher prevalence of *H*. *pylori* infection in sexual partners of *H*. *pylori*-infected subjects than in controls^9^.

Whilst most of the available literature on risk factors for *H*. *pylori* infection have focused on environmental and lifestyle factors (e.g. smoking and diet), an increasing body of evidence for the role of genetic factors in susceptibility to *H*. *pylori* infection has recently emerged^10,11^. One genetically determined trait with known polymorphic expression between individuals and populations that has attracted interest as potential risk factors for *H*. *pylori* infection is ABO blood group^12^. This premise was developed from previous studies showing a higher frequency of blood group O amongst patients with duodenal ulcer^13^. Since duodenal ulcer disease is associated with antral *H*. *pylori* infection in 90–100% of cases, blood group O might also be a risk factor for acquiring *H*. *pylori* infection. ABO blood groups have also been investigated as risk factors for *H*. *pylori* associated gastric cancer, however there are conflicting studies due to multiple confounding effects^14^. Since the discovery of the ABO blood group, there has been an ongoing interest in the potential role of blood groups in infectious disease. Blood group antigens are receptors for toxins, parasites, and bacteria, where they can facilitate colonization or invasion or evade host clearance mechanisms^15^. Previous studies demonstrated that blood-group antigen-binding adhesion (BabA) mediate adherence of *H*. *pylori* to human Lewis^b^ (α-1,3/4-difucosylated) blood-group antigens on gastric epithelial cells^16,17^.

During the last few decades, evidence for the role of ABO groups as potential risk factors for *H*. *pylori* infection has emerged from animal model studies and epidemiological data^18–20^. *H*. *pylori* infection was found in some studies to be positively associated with O blood group^18,21^, whilst others found no correlations^19,20,22,23^.

Meta-analysis is a well-established method that pools data from smaller inconclusive studies to provide greater statistical power^24^. Therefore, the current systematic review and meta-analysis of the relevant epidemiologic literature aims to quantify the association between ABO blood group and *H*. *pylori* infection status.

## Methods

### Literature search and study selection

Comprehensive inclusion and exclusion criteria were pre-defined and then used to objectively identify relevant epidemiological studies in MEDLINE/PubMed databases (up to October 2017). The studies had to satisfy the following requirements: (i) published in English; (ii) cross-sectional, cohort, case–control study, or randomized controlled trial; (iii) ABO blood groups were defined using the slide method, tube test, microplate technology, column/gel centrifugation, surface imprinting of erythrocytes, use of synthetic and natural receptors for ABO blood group sensors, chromatography and filtration paper based diagnostics, and any other molecular blood typing methods, (iv) *H*. *pylori* infection was determined using a stool antigen test, serum *H*. *pylori*-specific IgG using ELISA, ^13^C- or ^14^C-labelled urea breath test, antral biopsy urease test, microbiological culture methods or histological identification of organisms, hematoxylin and eosin (HE) staining, or Giemsa staining. Reviews, letters, correspondences, editorials, and case reports were excluded from analysis. Two researchers (ZC and BT) independently evaluated the eligibility of studies using a three-step process starting with examination of titles, then paper abstracts, and ultimately full-texts. References were also reviewed to determine new qualified studies. Relevant papers were imported using the EndNote X7 software (Thomson Reuters Corporation, New York, NY, USA).

### Study quality assessment

The Newcastle–Ottawa quality assessment scale (NOS) was adapted to observational studies and used to evaluate their quality^25^. Three factors were considered when determining a quality score: selection of the study groups; comparability of groups; and ascertainment of the exposure and outcomes. High quality studies were considered to have a score of 7 or greater out of 10 for cross-sectional and cohort studies.

### Statistical analysis

Comprehensive Meta-Analysis (CMA; version 3) was used to analyze reported odds ratios (ORs) and 95% confidence intervals (CIs) for the association between ABO blood groups and *H*. *pylori* infection status. Studies with reported raw data required calculation to retrieve ORs and CIs. ABO blood group outcomes were used for primary analysis. Relative risk was measured as OR in all studies and adjusted ORs were preferred to crude ORs. A random study effects model, accounting for heterogeneity, was used to combine study individual effect estimates. Cochran Q and I^2^ statistics were used to evaluate heterogeneity. Cochran Q with a P value <0.10 was considered to be statistically significant for heterogeneity. A value >50% for I^2^ was considered to have severe heterogeneity^26^. Asymmetry in funnel plots of study ORs versus the standard error of logarithm of these ORs were used to assess publication/selection bias. Egger’s regression asymmetry test was used to evaluate the degree of asymmetry^27^. Sensitivity analyses were performed to assess the impact and influence of individual studies on overall pooled estimates^28,29^.

Subgroup analyses were completed subsequent to the primary analysis according to study quality (low or high), study design and study area (developed and developing). The current meta-analysis and review was performed in accordance to the Preferred Reporting Items for Systematic reviews and Meta-Analyses (PRISMA)^30^ and Meta-analysis of Observational Studies in Epidemiology group (MOOSE)^31^.

## Results

### Study characteristics

**Figure 1:**
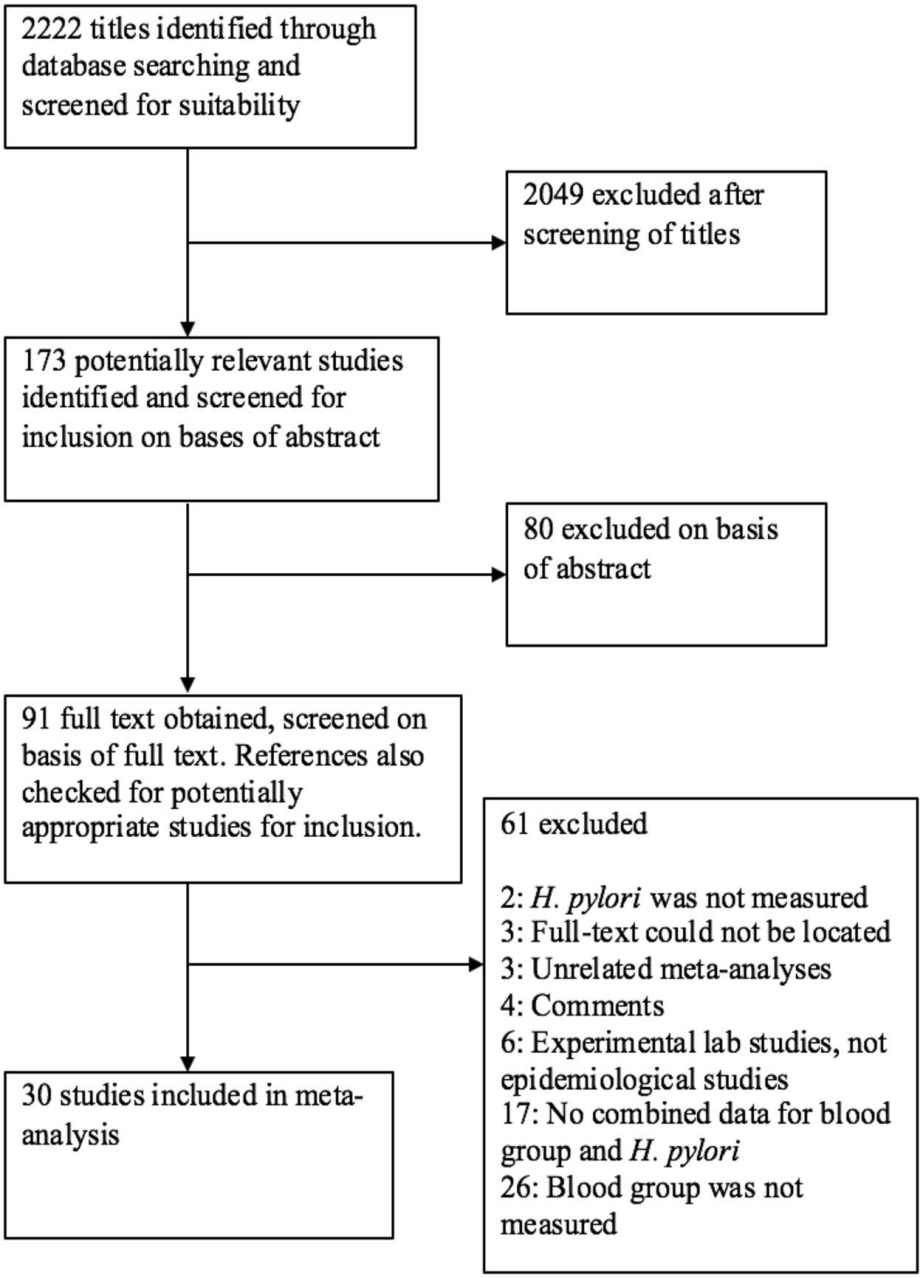
Flowchart of study selection process.

The comprehensive search of relevant literature yielded 2222 potentially relevant studies. Full texts of 91 papers were retrieved after title and abstract examination steps (Fig. 1). Of the 91 papers, 30 were considered eligible and presented relevant data for the meta-analysis (Fig. 1).

Of the 30 eligible studies, twenty-seven were cross-sectional in, two were cohort studies, and one used a randomized control trial design. The majority of the studies included adults aged between 18 and 74 years; only two studies were performed in children under 15 years old. The 30 studies included a total of 12,708 individuals (Table 1).

**Table 1:** Summary characteristics of studies included in the meta-analysis.

First Author, Year	Country	Study Design	Outcome Measures	Exposure Measures	Type of Population	Country Development	Number of Participants	NOS
Niv, 1996	Israel	Cross-sectional	ELISA, antral biopsy urease, Giemsa	Blood test	Adults	Developing	187	7
Valliani, 2013	Pakistan	Cross-sectional	Histopathology	Anti-sera on slide method	Adults	Developing	93	6
Umlauft, 1996	Austria	Cross-sectional	HE Staining	Blood test	Adults	Developed	384	7
Rasmi,2011	Iran	Cross-sectional	ELISA	Hemagluttination	Adults	Developing	151	6
Petrovic, 2011	Serbia	Cross-sectional	Urea breath test	Hemagluttination	Adults	Developing	227	6
Kuyvenhoven, 1999	Netherlands	Cross-sectional	ELISA, antral biopsy urease, Giemsa	Hemagluttination	Adults	Developed	135	8
Klaamas, 1997	Estonia	Cross-sectional	ELISA	Blood test	Adults	Developed	159	8
Yei, 2005	Taiwan	Cross-sectional	Urea breath test	Antibody detection	N/A	Developing	230	7
Loffeld, 1991	N/A	Cross-sectional	ELISA	Blood test	Adults	N/A	402	7
Heneghan, 1998	Ireland	Cross-sectional	ELISA. HE Stain, Giemsa Stain	Hemagluttination	Adults	Developed	198	7
deMattos, 2002	Brazil	Cross-sectional	Urea breath test	Hemagluttination	Adults	Developing	120	7
Turkolmez, 2007	Turkey	Cross-sectional	Urea breath test	N/A	Adults	Developing	509	7
Robertson, 2003	Australia	Cohort	Rapid whole blood test, antibodies	Automated blood group analyser	Adults	Developed	499	8
Bhuiyan, 2009	Bangladesh	Cohort	ELISA, Stool antigen test	Hemagluttination	Children	Developing	234	7
Henrikkson, 1993	Sweden	RCT	Gastric juice cultivation	Plasma gastrin	Adults	Developed	42	N/A
Jafarzadeh, 2007	Iran	Cross-sectional	ELISA	Hemagluttination	Children	Developing	386	8
Kanbay, 2005	Turkey	Cross-sectional	Immulite analyzer	Antigen typing, ELISA	Adults	Developing	540	8
Ansari, 2015	Pakistan	Cross-sectional	PCR targetting, 16sRNA	ABO monoclonal reagent	Adults	Developing	259	7
Keller, 2002	Germany	Cross-sectional	ELISA	Hemagluttination	Adults	Developed	330	7
Bayan, 2008	Turkey	Cross-sectional	HE Staining	Gel Centrifugation	Adults	Developing	364	7
Oberhuber, 1997	Austria	Cross-sectional	Warthin-Starry Stain	Immunostaining	Adults	Developed	184	7
Wu, 2003	Taiwan	Cross-sectional	ELISA	Blood test	Adults	Developing	425	8
Tadesse, 2014	Ethiopia	Cross-sectional	ELISA	Hemagluttination	Adults	Developing	408	7
Moges, 2006	Ethiopia	Cross-sectional	Hexagon serology	Hemagluttination	Adults	Developing	215	7
Klaamas, 1999	Estonia	Cross-sectional	ELISA	Antibody detection	Adults	Developed	450	7
Lin, 1998	Taiwan	Cross-sectional	Gram Staining, Culture	N/A	Children/Adults	Developing	775	7
Nakao, 2011	Japan	Cross-sectional	ELISA	Single nucleotide polymorphism	Adults	Developed	1406	8
Risch, 2010	USA	Cross-sectional	ELISA	Hemagluttination	Adults	Developed	1063	7
Rizzato, 2013	Venezuela	Cross-sectional	Reverse Hybridization	Blood test	Adults	Developing	2062	7
Hook-Nikanne,1990	Finland	Cross-sectional	Immunoassay test	Blood test	Adults	Developed	271	7

*H*. *pylori* was defined using an enzyme-linked immunosorbent assay (ELISA) in ten studies, histological analysis of hematoxylin and eosin (HE) stained gastric tissue sections in two studies, a urea breath test in four studies, and a combination of ELISA, antral biopsy urease test, and Giemsa staining of histological sections in two studies. The remaining 12 studies used various other techniques to define *H*. *pylori*. The majority of the included studies determined ABO type via serum hemagglutination methods. These methods are sufficiently standard and widely used in large-scale blood banking (Table 1).

### Quality assessment

Of the 30 eligible studies, 26 were considered to be of higher methodological quality (score ≥ 7) and 3 were considered to be of lower quality due to an insufficient characterization of control populations, failure to report response rates, or lack of adjustment for confounding variables. In a range of 6–8, the median overall score was 7, which demonstrates the studies to be of generally high quality (Table 1).

### Association between O Blood groups and *H*. *pylori* infection

**Figure 2:**
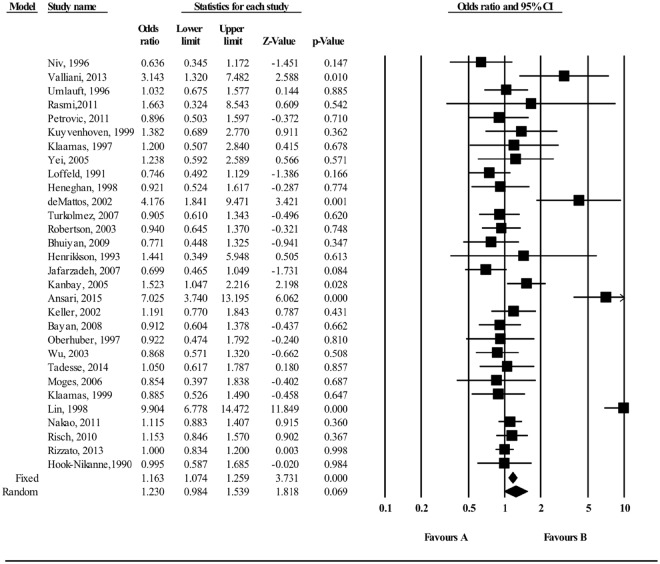
Meta-analysis of the association between O blood group and *H*. *pylori* infection. For each study, the box represents the fixed and random effects odds ratio and the line the 95% confidence intervals. The size of each box indicates the relative weight of each study in the meta-analysis. Test for overall fixed and random effects: z = 3.731, P < 0.001, I^2^ = 85.52, P < 0.001; z = 1.818, P = 0.069, respectively.

The meta-analysis of findings from 30 studies on the association between O blood group and *H*. *pylori* infection (Fig. 2) demonstrated an increased odds of *H*. *pylori* infection compared to non-O blood group (OR; 1.163; 95% CI; 1.074–1.259, P < 0.001). Sixteen of the 30 studies independently reported an OR greater than or equal to 1. Although most of the studies found a positive association between O blood group and *H*. *pylori* (Fig. 2), there was substantial heterogeneity (I^2^ = 85.52%) in estimated effect size between the studies suggesting that systematic effect size variability was unaccounted for. Thus, factors associated with the studies themselves (e.g., quality of the study, study design) and participant characteristics (e.g. place of residence or age) may have moderated the overall results. We therefore conducted subgroup analyses to determine the extent to which these variables moderated the overall results. In a subgroup analysis, O blood group was also significantly associated with *H*. *pylori* infection (OR = 1.268: 95% CI 1.141–1.409, P < 0.001) when the data pool was restricted to seventeen studies that were conducted in a developing country, whilst the pooled OR for the twelve studies conducted in a developed country was weaker and not statistically significant (OR = 1.071, 95% CI; 0.944–1.214, P = 0.984; Fig. S1). Findings did not substantially change when the analysis was concentrated on the 26 high-quality studies (OR = 1.157, 95% CI 1.067–1.254; P < 0.01; Fig. S2).

**Figure 3:**
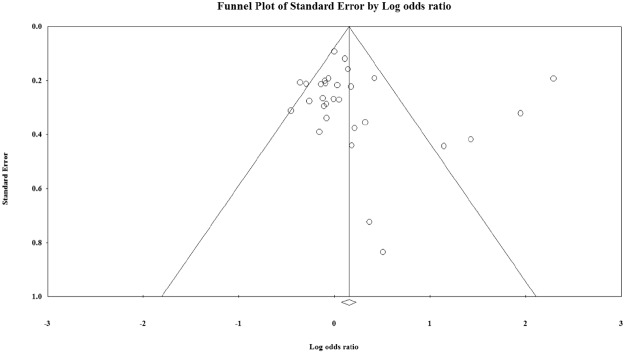
Funnel plot of standard error by log odds ration for the association between O blood group and *H*. *pylori* infection. Egger’s test; b = 0.80198, P = 0.48672.

The 27 cross-sectional studies resulted in a pooled OR of 1.185 (95% CI; 1.091–1.286; P < 0.01), Fig. S3). Publication bias did not appear to play a role in the association between blood group O and *H*. *pylori* infection since no clear deviations from symmetry was observed in the funnel plot (Fig. 3). The Egger regression asymmetry test also demonstrated no statistically significant publication biases (Egger’s test; b = 0.802, P = 0.486). Furthermore; robustness of the observed outcome was investigated by sequentially removing each study and re-analyzing the data sets and no study was shown substantially alter the pooled OR estimate (Supplement Fig. S4).

### Association between B Blood group and *H*. *pylori* infection

**Figure 4:**
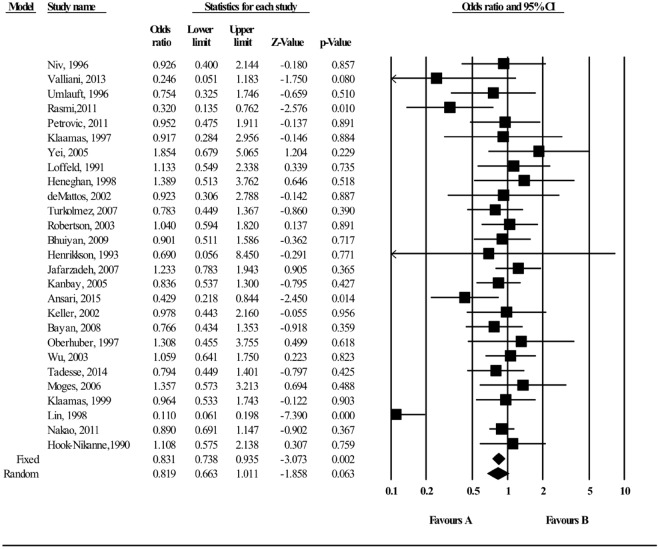
Meta-analysis of the association between B blood group and *H*. *pylori* infection. Test for overall fixed and random effects: z = −3.073, P = 0.002, I^2^ = 62.31, P < 0.001; z = −1.858, P = 0.063, respectively.

**Figure 5:**
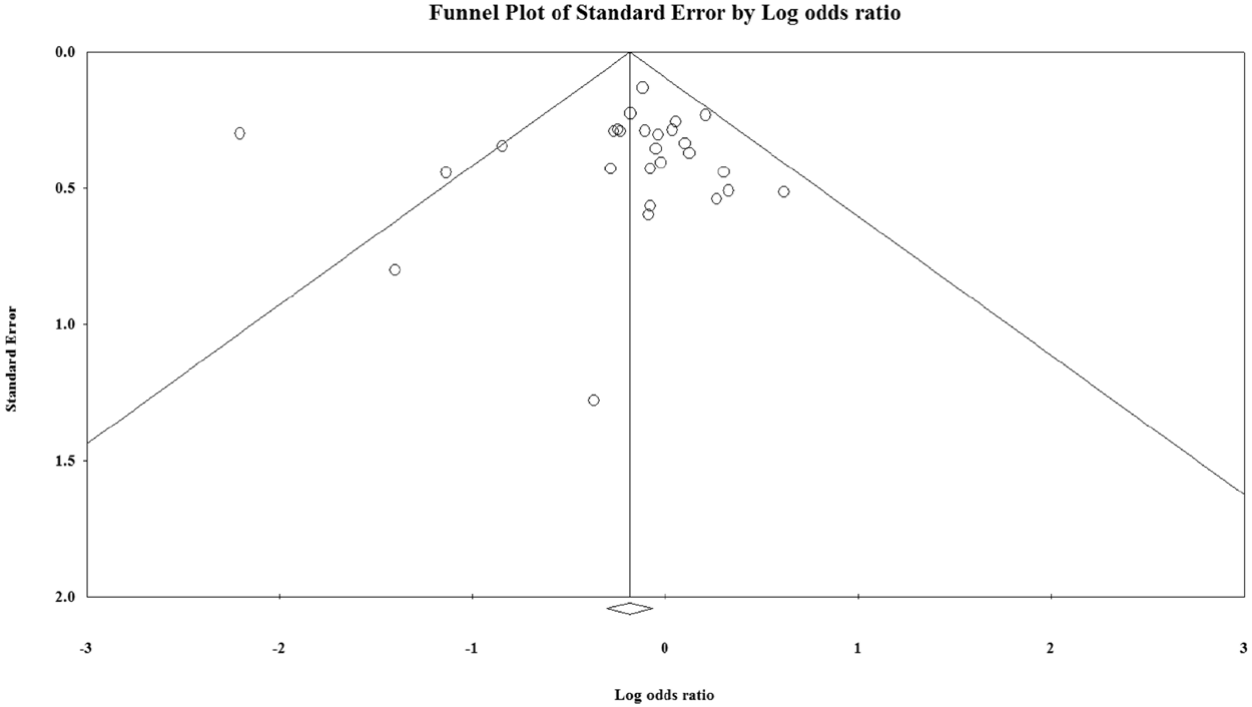
Funnel plot of standard error by log odds ration for the association between B blood group and *H*. *pylori* infection. Egger’s test; b = −0.221, P = 0.79117.

The meta-analysis of findings from 27 studies on the association between B blood group and *H*. *pylori* infection (Fig. 4) demonstrated a slightly decreased odds of *H*. *pylori* infection (OR = 0.831 (95% CI; 0.738–0.935, p = 0.002). Eighteen of the 27 studies independently reported an OR less than 1, and evidence of substantial heterogeneity was observed (I^2^ = 62.31). A subgroup analysis of sixteen studies from a developing country produced a summary risk estimate of 0.741 (95% CI 0.633–0.868, P < 0.001, Fig. S5). Further stratified analysis by study quality and study design did not materially alter the size of pooled estimate: with 23 high-quality studies (OR = 0.850, 95% CI 0.752–0.959; P = 0.009; Fig. S6), and 24 cross-sectional studies (OR of 0.819 (95% CI; 0.723–0.927; P = 0.002; Fig. S7). There was no evidence of publication bias on both visual inspections of the funnel plot (Fig. 5) and from the result of Egger’s test (Egger’s test; b = −0.221, P = 0.792). Furthermore, a sensitivity analyses was completed by sequentially removing each study and re-analyzing the data sets provided a nearly identical risk estimate (Supplement Fig. S8).

### Association between AB Blood group and *H*. *pylori* infection

**Figure 6:**
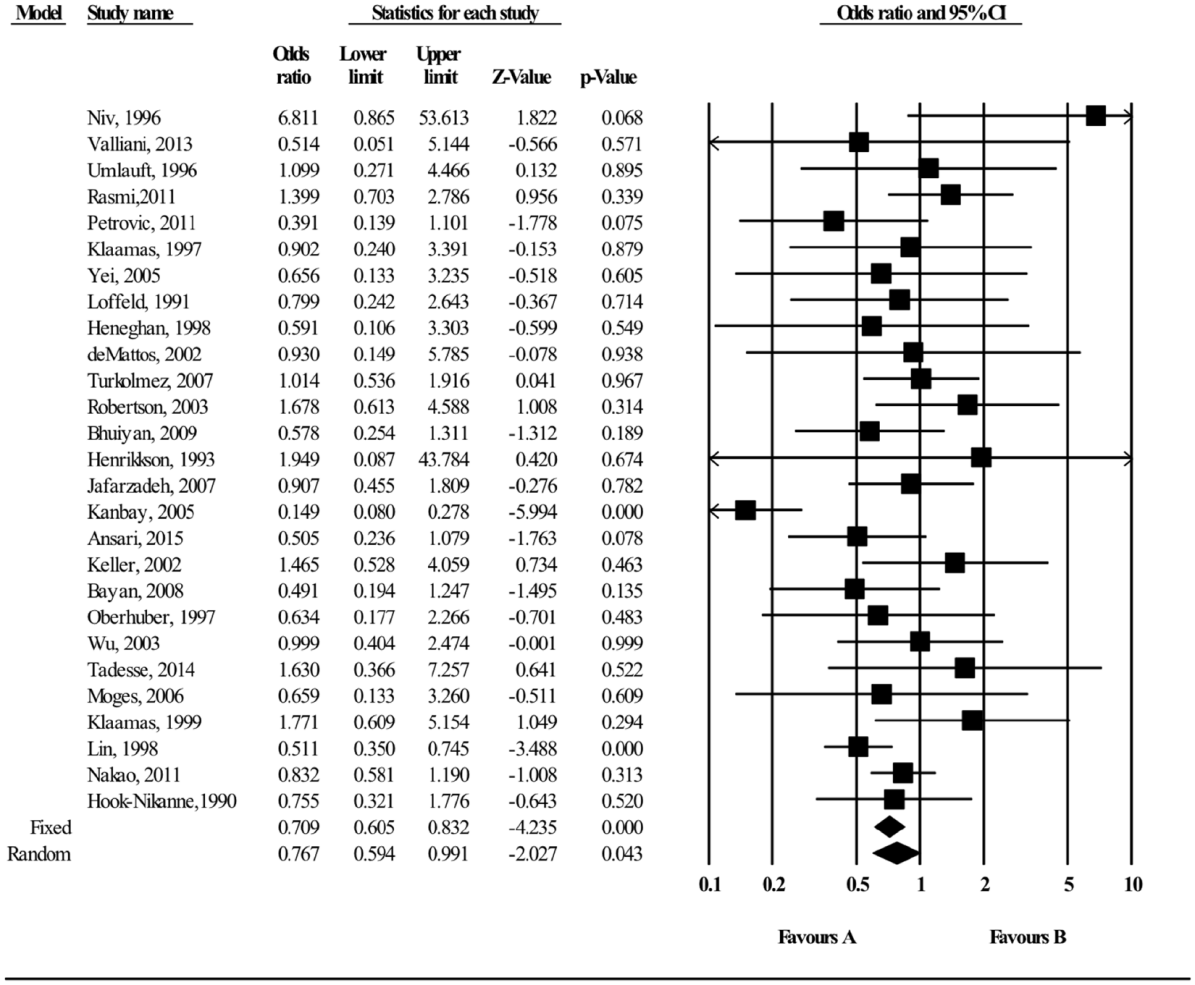
Meta-analysis of the association between AB blood group and *H*. *pylori* infection. Test for overall fixed and random effects: z = −4.235, P < 0.001, I^2^ = 49.27, P = 0.002; z = −2.027, P = 0.043, respectively.

**Figure 7:**
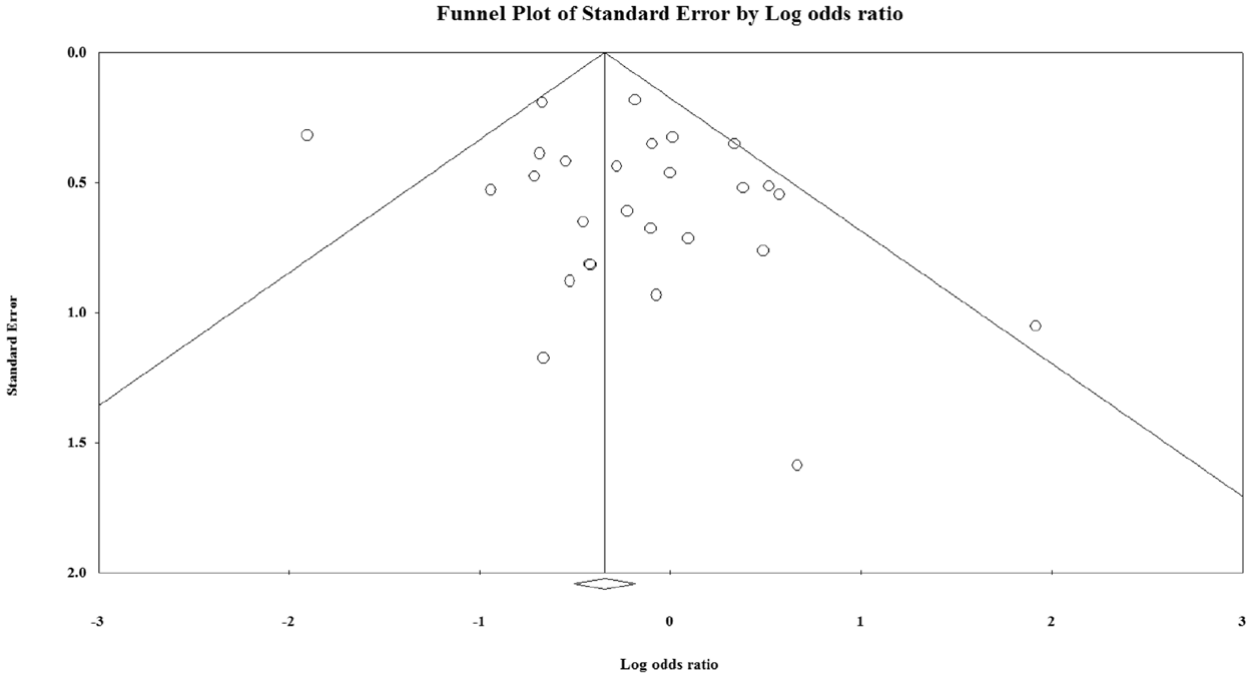
Funnel plot of standard error by log odds ration for the association between AB blood group and *H*. *pylori* infection. Egger’s test; b = 0.77514, P = 0.17212.

The meta-analysis of findings from 27 studies on the association between AB blood group and *H*. *pylori* infection (Fig. 6) demonstrated decreased odds of *H*. *pylori* infection (OR:0.709; 95% CI; 0.605–0.832, p < 0.000), and there was moderate heterogeneity across studies (I^2^ = 49.2%). The findings from the subgroup analyses based on different methodological criteria did not materially alter the size of pooled estimates (OR:0. 601 (95% CI 0.492–0.735, P < 0.001; 16 studies from developing country; Fig. S9, OR = 0.692, 95% CI 0.586–0.816; P < 0.01 in 23 high quality studies; Fig. S10, and OR = 0.697; 95% CI; 0.591–0.822; P < 0.01 in 24 cross sectional studies Fig. S11). No significant deviations from symmetry were observed in the funnel plot that might suggest evidence of publication bias (Fig. 7). No statistical significant publication bias was also shown by the Egger regression asymmetry test (Egger’s test; b = 0.775, P = 0.172). Robustness of the observed outcome was investigated by sequentially removing each study and re-analyzing the data sets leading to no substantial alteration to the pooled OR estimate by any single study (Fig. S12).

### Association between A Blood groups and *H*. *pylori* infection

**Figure 8:**
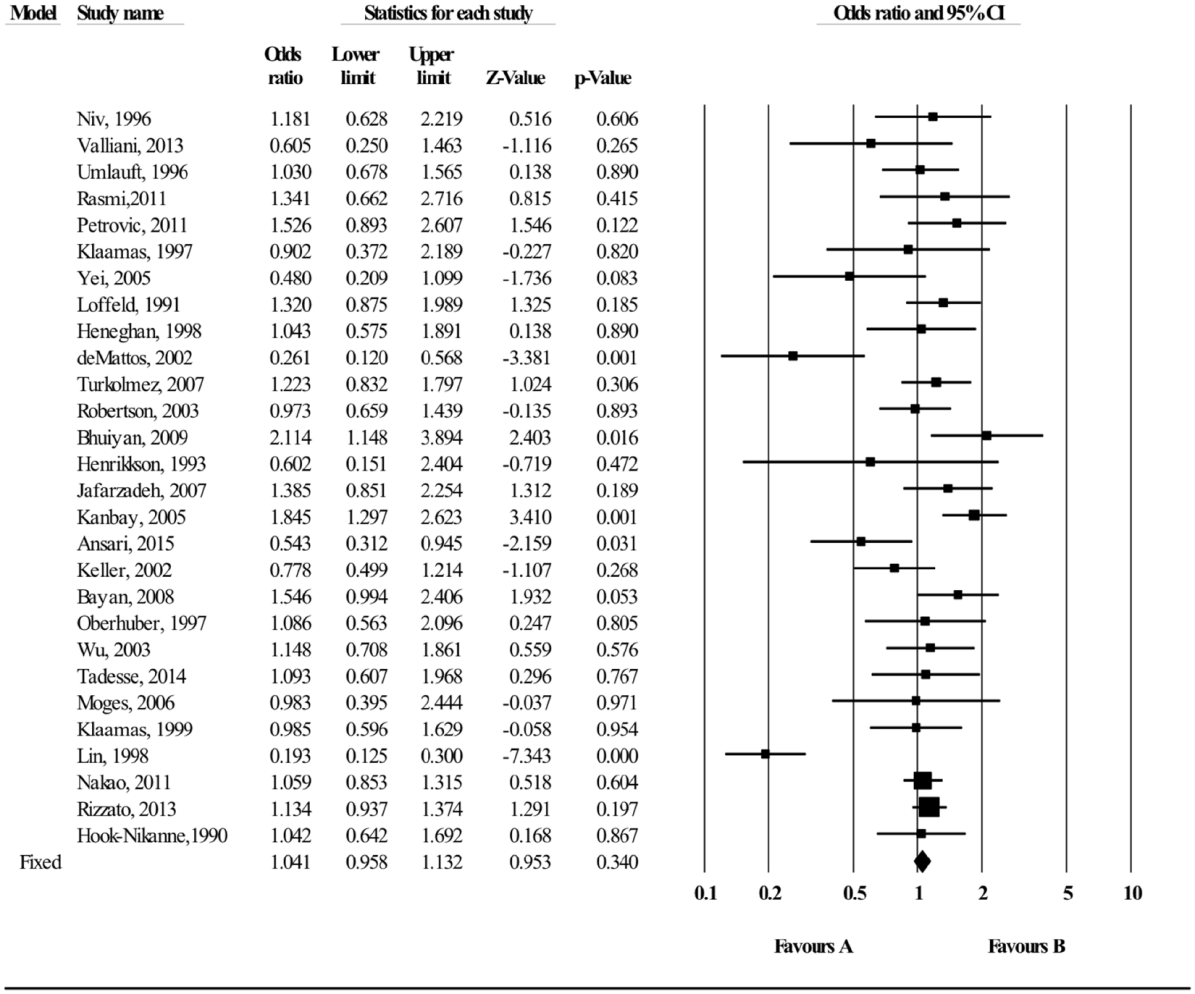
Meta-analysis of the association between A blood group and *H*. *pylori* infection. Test for overall fixed and random effects: z = 0.953, P = 0.340, I^2^ = 74.68, P < 0.001; z = −0.213, P = 0.831, respectively.

Figure 8 shows an overall pooled OR for the association between A blood group and *H*. *pylori* infection. No evidence was found suggesting that individuals with A blood group significantly associated with risk of *H*. *pylori* infection (OR = 1.041; 95% CI; 0.958–1.132, p = 0.340). There was also evidence of substantial heterogeneity (I^2^ = 74.68), however, symmetry was observed in the funnel plot, indicating no publication bias (Fig. 8). Furthermore, No statistical significant publication bias was also shown by the Egger regression asymmetry test (Egger’s test; b = 0.80198, P = 0.486). A subgroup analysis analyses based on different methodological criteria did not materially alter the size of pooled estimates (Data not shown). No single study substantially altered the pooled OR estimates in the sensitivity analysis (Data not shown).

### Association between Rh Blood group and *H*. *pylori* infection

**Figure 9:**
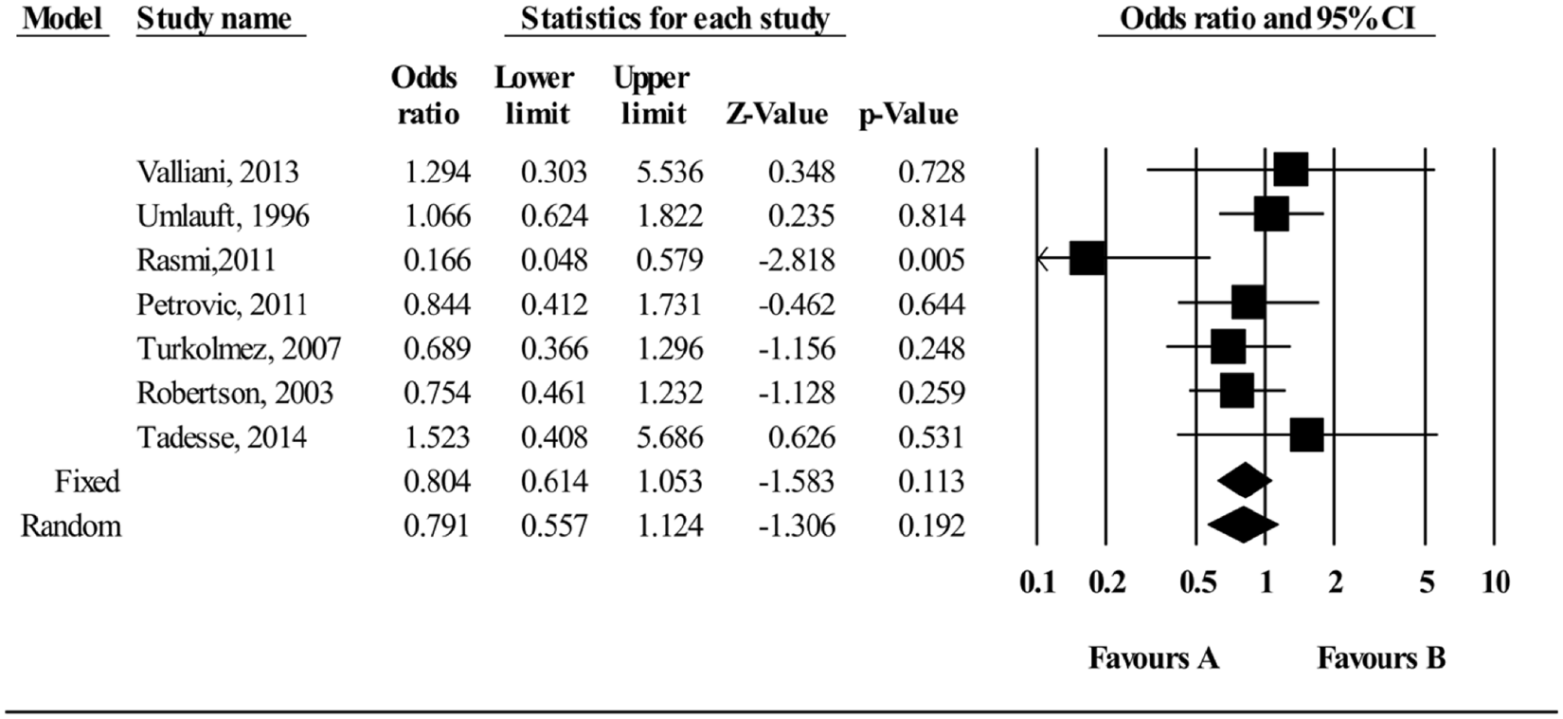
Meta-analysis of the association between Rh blood group and *H*. *pylori* infection. Test for overall fixed effects and random effects: z = −1.583, P = 0.113, I^2^ = 31.999, P = 0.184; z = −1.306, P = 0.192, respectively.

Figure 9 shows an overall pooled OR for the association between Rh blood group and *H*. *pylori* infection. Overall, Rh positive was associated with non-significant reduction risk in *H*. *pylori* infection (pooled OR 0.804 (95% CI 0.614–1.053). There was evidence of heterogeneity (P < 0.0001), with a moderate proportion of the total variation in the estimated effect due to between-study heterogeneity (I^2^ = 31.9%).

### Association between secretor status and *H*. *pylori* infection

**Figure 10:**
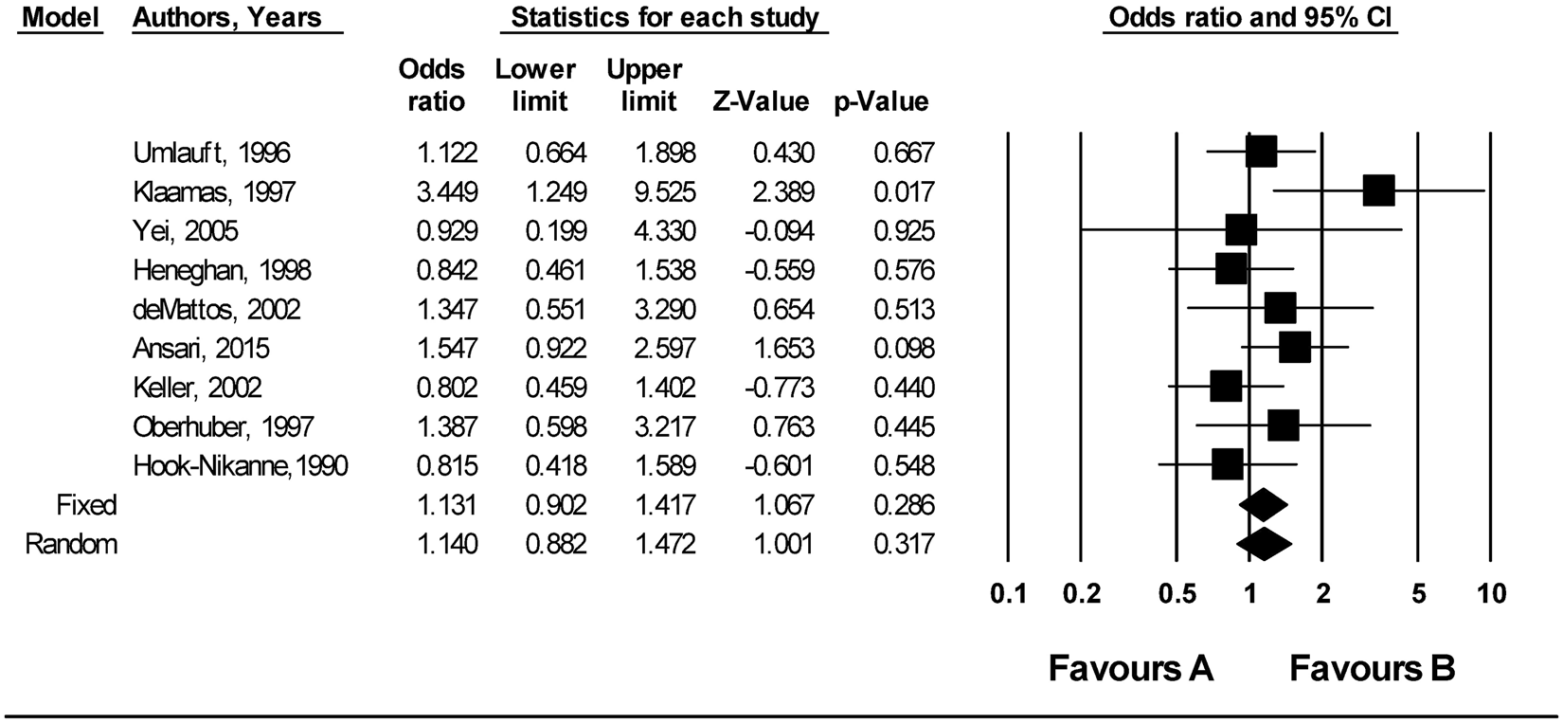
Meta-analysis of the association between secretor status and *H*. *pylori* infection. Test for overall fixed and random effects: z = 1.067, P = 0.286, z = 1.001, P = 0.317, respectively. I^2^ = 18.157, P = 0.184.

Nine studies reported secretor status (secretors belong to Lewis (Le)(a-b+) and those who are non- secretors to Le (a-b−) histo-blood group phonotype). The pooled analysis showed a slightly higher odds of *H*. *pylori* infection among secretors compared to non-secretors (OR = 1.140 (95% CI; 0.882–1.472, p = 0.317) (Fig. 10). Five of the 9 studies individually reported an OR greater than 1, and evidence of minimum heterogeneity was observed (I^2^ = 18.157, P = 0.184.). There was no evidence of publication bias on both visual inspections of the funnel plot (Fig. S13) and from the result of Egger’s test (Egger’s test; b = 1.0672, P = 0.441). Sensitivity analyses indicated that there was little evidence to suggest that the pooled odds ratio was influenced by any single study (Data not shown).

## Discussion

We believe that this is the first meta-analysis that investigates the association between ABO blood group and *H*. *pylori* infection. Our meta-analysis of 30 eligible studies consisting of more than 12,000 individuals showed O blood group to be associated with an estimated 16.3% increase in probability of *H*. *pylori* infection than non-O blood group, which was highly statistically significant. Whilst, blood group B and AB were found to be associated with an estimated 17% and 29% reduction in odds of *H*. *pylori* infection compared with non-B, non-AB, respectively, also highly statistically significant. The results did not vary much from the overall association when observing study area (developing and developed countries), study quality, or study design. No evidence of publication bias was found on both visual inspections of the funnel plot and from the result of Egger’s test. However, there was moderate to substantial heterogeneity in the estimated effect size between studies suggesting that systematic effect size variability was unaccounted for. Thus, factors associated with the studies themselves (e.g. quality of the study, study design) and participant characteristics (e.g. place of residence, age) may have moderated the overall results. Our subgroup analysis, however, did not materially alter the size of pooled estimates. We observed a limited change in association regardless of study quality, study design or study area. Our meta-analysis found no evidence that A and Rh blood group groups significantly associated with risk of *H*. *pylori* infection. We attempted to perform potential disease subgroup analyses, however there was an insufficient number of epidemiological papers to see the relationship between *H*. *pylori* and ABO amongst PUD or gastric cancer compared to NUD. Further studies are needed in this area in order to clarify the impact of blood groups on disease risk.

Our findings should be interpreted in light of the following limitations. First, caution is warranted in the overall estimates provided in this meta-analysis, as there was significant heterogeneity. The lack of any explanation for this heterogeneity, apart from method of exposure and outcome assessments and possibly variation in the populations studied, suggests that methodological variations that we were not able to test may have been important. An alternative approach would be individual participant meta-analysis, as it would provide some consistency across the potentially mediating characteristics. Although, a high statistical heterogeneity across the studies still limiting the interpretation of the pooled estimate, the subgroup analysis, as well as sensitivity analysis, showed that results from our meta-analysis was robust. Second, because this meta-analysis was based on observational studies, our results were prone to selection bias inherent in the included studies. In addition, the potential confounding from other risk factors could not be ruled out, especially when considering that some studies only reported crude risk estimated without adjustments. Third, by narrowing our studies to only those of the English language, it is possible that some suitable studies published other languages may have been missed. Although, we did not find evidence of publication bias on both visual inspections of the funnel plot and from the result of Egger’s test, it might still be possible that some unpublished studies may have been missed.

A major strength of this review was the comprehensive search strategy in the PubMed database used to identify relevant studies. Furthermore, to reduce the possibility of missing eligible studies, reference lists of retrieved papers were checked. Additionally, study eligibility was meticulously assessed by an independent reviewer and further evaluated by an additional independent reviewer when necessary. Most of the eligible studies were considered of high methodological quality and outcomes were consistent after excluding studies of lower quality. Specific inclusion criteria were used regarding the method of ascertainment and other facets, effectively reducing reporting bias that may emerge from use of a subjective self-reported method. Our eligibility criteria required that the outcome of interest, *H*. *pylori* infection, be objectively measured. The majority of studies used ELISA tests to inexpensively and noninvasively detect anti-*H*. *pylori* IgG antibodies to define *H*. *pylori* infection. For ABO phenotype determination, most of the included studies used standard hemagglutination methods that have been in clinical use for the major blood types for a century^32^ and on which blood banking relies. Furthermore, the meta-analysis exhibits high statistical power through its use of 30 studies and over 12,000 individuals.

The underlying mechanism for the relationship between ABO blood group and *H*. *pylori* infection has been proposed from previous biochemical studies in ABO, Lewis and Secretor histo-blood system^17,33^. Individuals from O blood group carrying the secretor phenotype converts the precursor oligosaccharide type 1 into H antigen type 1 but due to the absence of GTA and or GTB glycosyltransferases they are unable to synthesize A or B antigens. Therefore, they convert H type 1 antigen into Le^b^ antigen in Lewis positive individuals (90% of the population). The high quantitative expression of this antigen in the gastric and duodenal mucosae of O blood group individuals carrying the secretor phenotype seems to increase the susceptibility to infection by *H*. *pylori* by providing a greater number of receptors for this microorganism^17^. The increase risk *of H*. *pylori* infection among individuals with O blood group, but not in non-O blood group (A, B, AB) in this meta-analysis fit with the aforementioned proposed biological mechanisms. Furthermore, the tendency of higher risk of *H*. *pylori* infection among secretor compared to non-secretor in our study fits with the early hypothesis proposed by Borén *et al*.^17^ in which bacteria choose to attach itself to the Lewis b antigen (Le^b^), which is rich in fucose and is expressed on the surface of the epithelial cells of the gastric mucosa^17^. Most of the included studies in our analysis did not provide secretor frequencies in each A, B, O individuals, however studies documented that O and Le (a-b+) phenotypes express a greater quantity of these fucosylated antigens in comparison with other groups which in turn predispose these carriers to *H*. *pylori* infection^17,34^, and influence the development of *H*. *pylori* associated complication. Although most epidemiological studies failed to report data on specific strains of *H*. *pylori* in relation to ABO group, experimental evidence has shown strain-dependent preference of BabA adhesin(s) in binding to MUC5AC glycoforms substituted with Le^b^ which may contribute interindividual variability in host-microbe interactions^35^.

In conclusion, this meta-analysis provides evidence that O blood group is associated with increased risk of *H*. *pylori* infection compared to non-O blood group. However, high quality cohort studies are necessary to ascertain causality, which may significantly aid in understanding the pathogenesis of *H*. *pylori* infection and discover potential preventative strategies. For instance, if the association between O blood type and *H*. *pylori* infection is confirmed by further controlled studies, O blood group status may be listed as one of *H*. *pylori* risk factors, and ABO blood typing may hence become part of a multifaceted strategy for *H*. *pylori* risk assessment.

## Electronic supplementary material

Supplementary information
