# Supplementary material for: Association Between ABO Blood Groups and Helicobacter pylori Infection: A Meta-Analysis
Source: Sci Rep. 2018 Dec 4;8:17604. doi: 10.1038/s41598-018-36006-x (PMC6279815; doi:10.1038/s41598-018-36006-x)
Supplement: Supplementary file 1 — Supplementary information [file 41598_2018_36006_MOESM1_ESM.pdf]

Association Between ABO Blood Groups and *Helicobacter pylori* Infection: A Meta-Analysis

Zakaria Chakrani[1], Karen Robinson[2], Bineyam Taye[1∞]

Online Supplemental Figures

Model	Group by Development	Study name	Odds ratio	Lower limit	Upper limit	Z-Value	p-Value
	Developed	Umlauft, 1996	1.032	0.675	1.577	0.144	0.885
	Developed	Kuyvenhoven, 1999	1.382	0.689	2.770	0.911	0.362
	Developed	Klaamas, 1997	1.200	0.507	2.840	0.415	0.678
	Developed	Heneghan, 1998	0.921	0.524	1.617	-0.287	0.774
	Developed	Robertson, 2003	0.940	0.645	1.370	-0.321	0.748
	Developed	Henrikkson, 1993	1.441	0.349	5.948	0.505	0.613
	Developed	Keller, 2002	1.191	0.770	1.843	0.787	0.431
	Developed	Oberhuber, 1997	0.922	0.474	1.792	-0.240	0.810
	Developed	Klaamas, 1999	0.885	0.526	1.490	-0.458	0.647
	Developed	Nakao, 2011	1.115	0.883	1.407	0.915	0.360
	Developed	Risch, 2010	1.153	0.846	1.570	0.902	0.367
	Developed	Hook-Nikanne,1990	0.995	0.587	1.685	-0.020	0.984
Fixed	Developed		1.071	0.944	1.214	1.067	0.286
Random	Developed		1.071	0.944	1.214	1.067	0.286
	Developing	Niv, 1996	0.636	0.345	1.172	-1.451	0.147
	Developing	Valliani, 2013	3.143	1.320	7.482	2.588	0.010
	Developing	Rasmi,2011	1.663	0.324	8.543	0.609	0.542
	Developing	Petrovic, 2011	0.896	0.503	1.597	-0.372	0.710
	Developing	Yei, 2005	1.238	0.592	2.589	0.566	0.571
	Developing	deMattos, 2002	4.176	1.841	9.471	3.421	0.001
	Developing	Turkolmez, 2007	0.905	0.610	1.343	-0.496	0.620
	Developing	Bhuiyan, 2009	0.771	0.448	1.325	-0.941	0.347
	Developing	Jafarzadeh, 2007	0.699	0.465	1.049	-1.731	0.084
	Developing	Kanbay, 2005	1.523	1.047	2.216	2.198	0.028
	Developing	Ansari, 2015	7.025	3.740	13.195	6.062	0.000
	Developing	Bayan, 2008	0.912	0.604	1.378	-0.437	0.662
	Developing	Wu, 2003	0.868	0.571	1.320	-0.662	0.508
	Developing	Tadesse, 2014	1.050	0.617	1.787	0.180	0.857
	Developing	Moges, 2006	0.854	0.397	1.838	-0.402	0.687
	Developing	Lin, 1998	9.904	6.778	14.472	11.849	0.000
	Developing	Rizzato, 2013	1.000	0.834	1.200	0.003	0.998
Fixed	Developing		1.268	1.141	1.409	4.418	0.000
Random	Developing		1.424	0.960	2.111	1.759	0.079
Fixed	Overall		1.182	1.091	1.282	4.071	0.000
Random	Overall		1.099	0.975	1.239	1.551	0.121

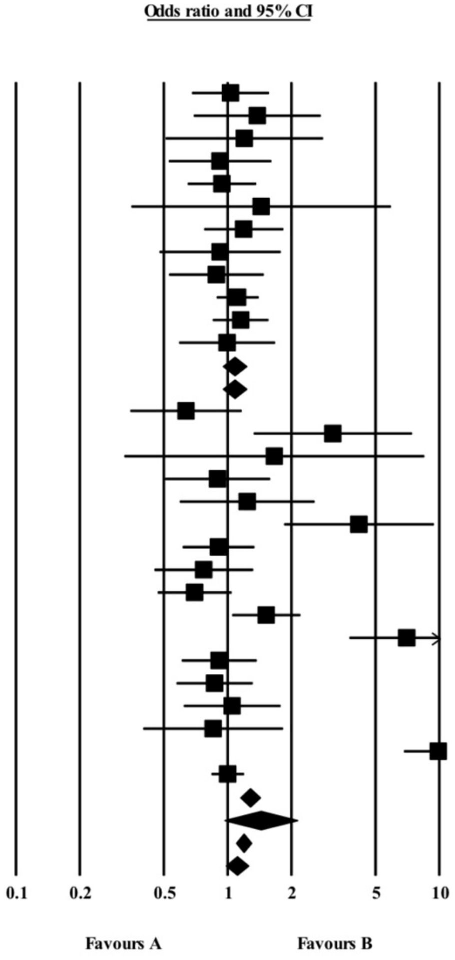

Fig. S1. Forest plot of the association between O blood group and H. pylori infection according to definition of income. Developed indicates studies completed in high income countries and developing indicates studies completed in low income countries. For each study, the box represents the random effects odds ratio and the line the 95% confidence intervals. The size of each box indicates the relative weight of each study in the meta-analysis. Test for overall fixed effects: Developed, z = 1.067; P = 0.0286; Developing, z = 4.418; P < 0.001; Overall z = 4.071; P < 0.001.

Model	Group by Study Quality	Study name	Odds ratio	Lower limit	Upper limit	Z-Value	p-Value
	High	Niv, 1996	0.636	0.345	1.172	-1.451	0.147
	High	Umlauft, 1996	1.032	0.675	1.577	0.144	0.885
	High	Kuyvenhoven, 1999	1.382	0.689	2.770	0.911	0.362
	High	Klaamas, 1997	1.200	0.507	2.840	0.415	0.678
	High	Yei, 2005	1.238	0.592	2.589	0.566	0.571
	High	Loffeld, 1991	0.746	0.492	1.129	-1.386	0.166
	High	Heneghan, 1998	0.921	0.524	1.617	-0.287	0.774
	High	deMattos, 2002	4.176	1.841	9.471	3.421	0.001
	High	Turkolmez, 2007	0.905	0.610	1.343	-0.496	0.620
	High	Robertson, 2003	0.940	0.645	1.370	-0.321	0.748
	High	Bhuiyan, 2009	0.771	0.448	1.325	-0.941	0.347
	High	Jafarzadeh, 2007	0.699	0.465	1.049	-1.731	0.084
	High	Kanbay, 2005	1.523	1.047	2.216	2.198	0.028
	High	Ansari, 2015	7.025	3.740	13.195	6.062	0.000
	High	Keller, 2002	1.191	0.770	1.843	0.787	0.431
	High	Bayan, 2008	0.912	0.604	1.378	-0.437	0.662
	High	Oberhuber, 1997	0.922	0.474	1.792	-0.240	0.810
	High	Wu, 2003	0.868	0.571	1.320	-0.662	0.508
	High	Tadesse, 2014	1.050	0.617	1.787	0.180	0.857
	High	Moges, 2006	0.854	0.397	1.838	-0.402	0.687
	High	Klaamas, 1999	0.885	0.526	1.490	-0.458	0.647
	High	Lin, 1998	9.904	6.778	14.472	11.849	0.000
	High	Nakao, 2011	1.115	0.883	1.407	0.915	0.360
	High	Risch, 2010	1.153	0.846	1.570	0.902	0.367
	High	Rizzato, 2013	1.000	0.834	1.200	0.003	0.998
	High	Hook-Nikanne,1990	0.995	0.587	1.685	-0.020	0.984
Fixed	High		1.157	1.067	1.254	3.547	0.000
Random	High		1.204	0.950	1.525	1.534	0.125
	Low	Valliani, 2013	3.143	1.320	7.482	2.588	0.010
	Low	Rasmi,2011	1.663	0.324	8.543	0.609	0.542
	Low	Petrovic, 2011	0.896	0.503	1.597	-0.372	0.710
Fixed	Low		1.342	0.846	2.129	1.251	0.211
Random	Low		1.592	0.642	3.946	1.004	0.315
Fixed	Overall		1.162	1.073	1.258	3.709	0.000
Random	Overall		1.225	0.974	1.540	1.738	0.082

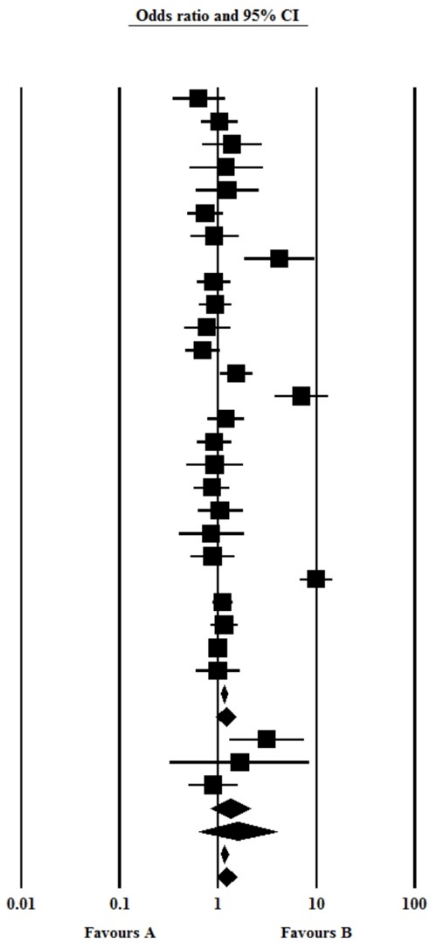

Fig. S2. Forest plot of the association between O blood group and *H. pylori* infection according to NOS quality scale. For each study, the box represents the random effects odds ratio and the line the 95% confidence intervals. The size of each box indicates the relative weight of each study in the meta-analysis. Test for overall fixed effects: High, z = 3.547; P < 0.001; Low, z = 1.251; P = 0.211; Overall, z = 3.709; P < 0.001.

Model	Group by Study Design	Study name	Statistics for each study				
			Odds ratio	Lower limit	Upper limit	Z-Value	p-Value
	Cohort	Robertson, 2003	0.940	0.645	1.370	-0.321	0.748
	Cohort	Bhuiyan, 2009	0.771	0.448	1.325	-0.941	0.347
Fixed	Cohort		0.881	0.647	1.201	-0.801	0.423
Random	Cohort		0.881	0.647	1.201	-0.801	0.423
	Cross-sectional	Niv, 1996	0.636	0.345	1.172	-1.451	0.147
	Cross-sectional	Valliani, 2013	3.143	1.320	7.482	2.588	0.010
	Cross-sectional	Umlauft, 1996	1.032	0.675	1.577	0.144	0.885
	Cross-sectional	Rasmi,2011	1.663	0.324	8.543	0.609	0.542
	Cross-sectional	Petrovic, 2011	0.896	0.503	1.597	-0.372	0.710
	Cross-sectional	Kuyvenhoven, 1999	1.382	0.689	2.770	0.911	0.362
	Cross-sectional	Klaamas, 1997	1.200	0.507	2.840	0.415	0.678
	Cross-sectional	Yei, 2005	1.238	0.592	2.589	0.566	0.571
	Cross-sectional	Loffeld, 1991	0.746	0.492	1.129	-1.386	0.166
	Cross-sectional	Heneghan, 1998	0.921	0.524	1.617	-0.287	0.774
	Cross-sectional	deMattos, 2002	4.176	1.841	9.471	3.421	0.001
	Cross-sectional	Turkolmez, 2007	0.905	0.610	1.343	-0.496	0.620
	Cross-sectional	Jafarzadeh, 2007	0.699	0.465	1.049	-1.731	0.084
	Cross-sectional	Kanbay, 2005	1.523	1.047	2.216	2.198	0.028
	Cross-sectional	Ansari, 2015	7.025	3.740	13.195	6.062	0.000
	Cross-sectional	Keller, 2002	1.191	0.770	1.843	0.787	0.431
	Cross-sectional	Bayan, 2008	0.912	0.604	1.378	-0.437	0.662
	Cross-sectional	Oberhuber, 1997	0.922	0.474	1.792	-0.240	0.810
	Cross-sectional	Wu, 2003	0.868	0.571	1.320	-0.662	0.508
	Cross-sectional	Tadesse, 2014	1.050	0.617	1.787	0.180	0.857
	Cross-sectional	Moges, 2006	0.854	0.397	1.838	-0.402	0.687
	Cross-sectional	Klaamas, 1999	0.885	0.526	1.490	-0.458	0.647
	Cross-sectional	Lin, 1998	9.904	6.778	14.472	11.849	0.000
	Cross-sectional	Nakao, 2011	1.115	0.883	1.407	0.915	0.360
	Cross-sectional	Risch, 2010	1.153	0.846	1.570	0.902	0.367
	Cross-sectional	Rizzato, 2013	1.000	0.834	1.200	0.003	0.998
	Cross-sectional	Hook-Nikanne,1990	0.995	0.587	1.685	-0.020	0.984
Fixed	Cross-sectional		1.185	1.091	1.286	4.050	0.000
Random	Cross-sectional		1.265	0.993	1.610	1.904	0.057
Fixed	Overall		1.162	1.073	1.258	3.709	0.000
Random	Overall		1.103	0.912	1.334	1.008	0.313

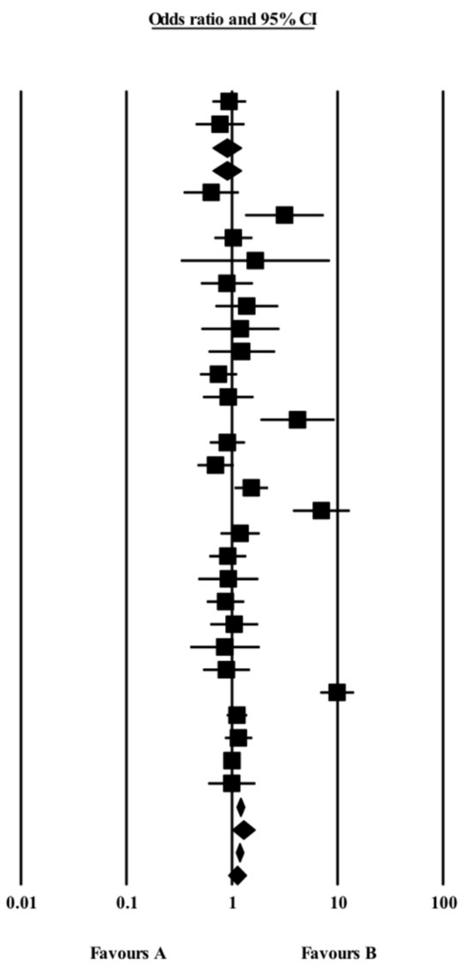

Fig. S3. Forest plot of the association between O blood group and H. pylori infection according to study design. For each study, the box represents the random effects odds ratio and the line the 95% confidence intervals. The size of each box indicates the relative weight of each study in the meta-analysis. Test for overall fixed effects: Cohort, z = -0.801; P = 0.423; Cross-sectional, z = 4.050; P < 0.001; Overall, z = 3.709; P < 0.001.

Study name	Statistics with study removed				
	Point	Lower limit	Upper limit	Z-Value	p-Value
Niv, 1996	1.175	1.085	1.273	3.953	0.000
Valliani, 2013	1.153	1.065	1.249	3.510	0.000
Umlauff, 1996	1.168	1.077	1.266	3.771	0.000
Rasmi,2011	1.162	1.073	1.258	3.706	0.000
Petrovic, 2011	1.169	1.079	1.266	3.818	0.000
Kuyvenhoven, 1999	1.160	1.071	1.256	3.651	0.000
Klaamas, 1997	1.162	1.074	1.259	3.709	0.000
Yei, 2005	1.162	1.073	1.258	3.692	0.000
Loffeld, 1991	1.182	1.091	1.282	4.071	0.000
Heneghan, 1998	1.168	1.078	1.266	3.810	0.000
deMattos, 2002	1.149	1.061	1.244	3.416	0.001
Turkolmez, 2007	1.175	1.084	1.274	3.910	0.000
Robertson, 2003	1.174	1.083	1.273	3.886	0.000
Bhuiyan, 2009	1.173	1.083	1.271	3.911	0.000
Henrikkson, 1993	1.162	1.073	1.258	3.709	0.000
Jafarzadeh, 2007	1.186	1.094	1.286	4.148	0.000
Kanbay, 2005	1.148	1.059	1.245	3.343	0.001
Ansari, 2015	1.130	1.043	1.224	2.993	0.003
Keller, 2002	1.162	1.072	1.259	3.649	0.000
Bayan, 2008	1.174	1.083	1.272	3.888	0.000
Oberhuber, 1997	1.167	1.077	1.264	3.787	0.000
Wu, 2003	1.175	1.084	1.274	3.927	0.000
Tadesse, 2014	1.165	1.076	1.263	3.746	0.000
Moges, 2006	1.167	1.077	1.263	3.793	0.000
Klaamas, 1999	1.170	1.080	1.268	3.846	0.000
Lin, 1998	1.055	0.972	1.144	1.285	0.199
Nakao, 2011	1.169	1.075	1.272	3.637	0.000
Risch, 2010	1.164	1.072	1.263	3.621	0.000
Rizzato, 2013	1.204	1.103	1.315	4.143	0.000
Hook-Nikanne,1990	1.167	1.077	1.264	3.777	0.000
	1.163	1.074	1.259	3.731	0.000

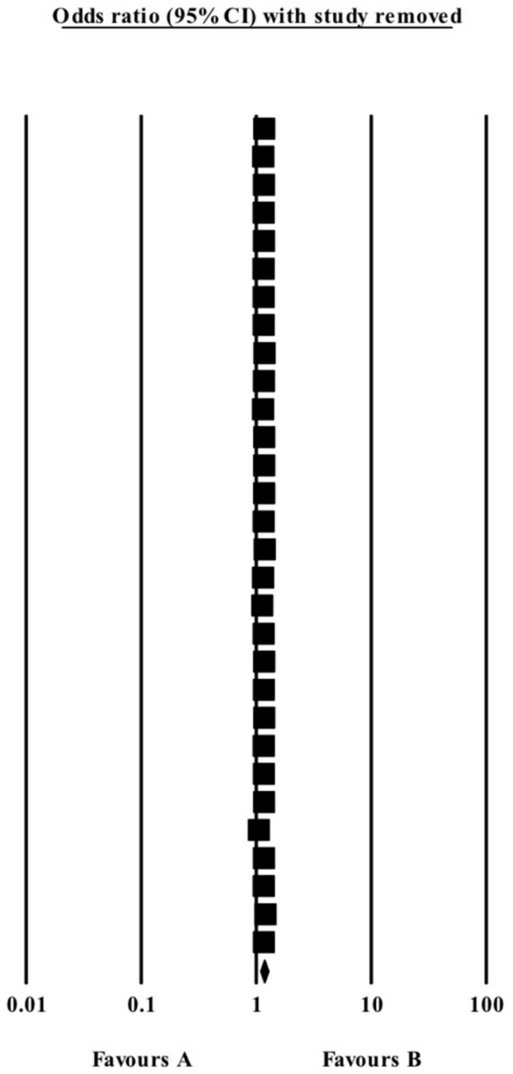

Fig. S4. Sensitivity analysis for O blood group and *H. pylori*. The vertical axis shows the omitted study. The box represents the random effects odds ratio and the line the 95% confidence intervals when the named study is omitted from the meta-analysis.

Model	Group by Development	Study name	Statistics for each study				
			Odds ratio	Lower limit	Upper limit	Z-Value	p-Value
	Developed	Umlauft, 1996	0.754	0.325	1.746	-0.659	0.510
	Developed	Klaamas, 1997	0.917	0.284	2.956	-0.146	0.884
	Developed	Heneghan, 1998	1.389	0.513	3.762	0.646	0.518
	Developed	Robertson, 2003	1.040	0.594	1.820	0.137	0.891
	Developed	Henrikkson, 1993	0.690	0.056	8.450	-0.291	0.771
	Developed	Keller, 2002	0.978	0.443	2.160	-0.055	0.956
	Developed	Oberhuber, 1997	1.308	0.455	3.755	0.499	0.618
	Developed	Klaamas, 1999	0.964	0.533	1.743	-0.122	0.903
	Developed	Nakao, 2011	0.890	0.691	1.147	-0.902	0.367
	Developed	Hook-Nikanne,1990	1.108	0.575	2.138	0.307	0.759
Fixed	Developed		0.949	0.790	1.141	-0.555	0.579
Random	Developed		0.949	0.790	1.141	-0.555	0.579
	Developing	Niv, 1996	0.926	0.400	2.144	-0.180	0.857
	Developing	Valliani, 2013	0.246	0.051	1.183	-1.750	0.080
	Developing	Rasmi,2011	0.320	0.135	0.762	-2.576	0.010
	Developing	Petrovic, 2011	0.952	0.475	1.911	-0.137	0.891
	Developing	Yei, 2005	1.854	0.679	5.065	1.204	0.229
	Developing	deMattos, 2002	0.923	0.306	2.788	-0.142	0.887
	Developing	Turkolmez, 2007	0.783	0.449	1.367	-0.860	0.390
	Developing	Bhuiyan, 2009	0.901	0.511	1.586	-0.362	0.717
	Developing	Jafarzadeh, 2007	1.233	0.783	1.943	0.905	0.365
	Developing	Kanbay, 2005	0.836	0.537	1.300	-0.795	0.427
	Developing	Ansari, 2015	0.429	0.218	0.844	-2.450	0.014
	Developing	Bayan, 2008	0.766	0.434	1.353	-0.918	0.359
	Developing	Wu, 2003	1.059	0.641	1.750	0.223	0.823
	Developing	Tadesse, 2014	0.794	0.449	1.401	-0.797	0.425
	Developing	Moges, 2006	1.357	0.573	3.213	0.694	0.488
	Developing	Lin, 1998	0.110	0.061	0.198	-7.390	0.000
Fixed	Developing		0.741	0.633	0.868	-3.709	0.000
Random	Developing		0.718	0.513	1.006	-1.924	0.054
Fixed	Overall		0.824	0.731	0.929	-3.171	0.002
Random	Overall		0.891	0.758	1.047	-1.407	0.159

Odds ratio and 95% CI

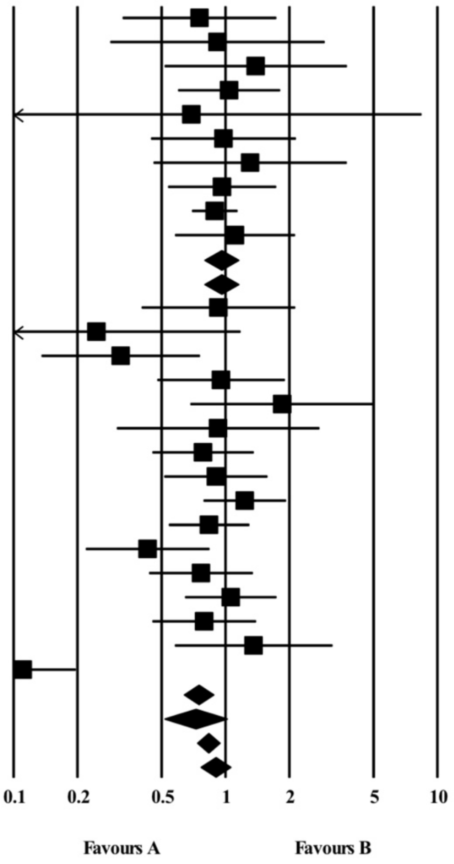

Fig. S5. Forest plot of the association between B blood group and H. pylori infection according to definition of income. Developed indicates studies completed in high income countries and developing indicates studies completed in low income countries. For each study, the box represents the random effects odds ratio and the line the 95% confidence intervals. The size of each box indicates the relative weight of each study in the meta-analysis. Test for overall fixed effects: Developed, z = -0.555; P = 0.579; Developing, z = -3.709; P < 0.001; Overall z = -3.171; P = 0.002.

Model	Group by Study Quality	Study name	Odds ratio	Lower limit	Upper limit	Z-Value	p-Value
	High	Niv, 1996	0.926	0.400	2.144	-0.180	0.857
	High	Umlauft, 1996	0.754	0.325	1.746	-0.659	0.510
	High	Klaamas, 1997	0.917	0.284	2.956	-0.146	0.884
	High	Yei, 2005	1.854	0.679	5.065	1.204	0.229
	High	Loffeld, 1991	1.133	0.549	2.338	0.339	0.735
	High	Heneghan, 1998	1.389	0.513	3.762	0.646	0.518
	High	deMattos, 2002	0.923	0.306	2.788	-0.142	0.887
	High	Turkolmez, 2007	0.783	0.449	1.367	-0.860	0.390
	High	Robertson, 2003	1.040	0.594	1.820	0.137	0.891
	High	Bhuiyan, 2009	0.901	0.511	1.586	-0.362	0.717
	High	Jafarzadeh, 2007	1.233	0.783	1.943	0.905	0.365
	High	Kanbay, 2005	0.836	0.537	1.300	-0.795	0.427
	High	Ansari, 2015	0.429	0.218	0.844	-2.450	0.014
	High	Keller, 2002	0.978	0.443	2.160	-0.055	0.956
	High	Bayan, 2008	0.766	0.434	1.353	-0.918	0.359
	High	Oberhuber, 1997	1.308	0.455	3.755	0.499	0.618
	High	Wu, 2003	1.059	0.641	1.750	0.223	0.823
	High	Tadesse, 2014	0.794	0.449	1.401	-0.797	0.425
	High	Moges, 2006	1.357	0.573	3.213	0.694	0.488
	High	Klaamas, 1999	0.964	0.533	1.743	-0.122	0.903
	High	Lin, 1998	0.110	0.061	0.198	-7.390	0.000
	High	Nakao, 2011	0.890	0.691	1.147	-0.902	0.367
	High	Hook-Nikanne,1990	1.108	0.575	2.138	0.307	0.759
Fixed	High		0.850	0.752	0.959	-2.627	0.009
Random	High		0.858	0.688	1.069	-1.367	0.172
	Low	Valliani, 2013	0.246	0.051	1.183	-1.750	0.080
	Low	Rasmi,2011	0.320	0.135	0.762	-2.576	0.010
	Low	Petrovic, 2011	0.952	0.475	1.911	-0.137	0.891
Fixed	Low		0.563	0.337	0.940	-2.198	0.028
Random	Low		0.483	0.200	1.162	-1.625	0.104
Fixed	Overall		0.831	0.738	0.936	-3.063	0.002
Random	Overall		0.829	0.669	1.026	-1.722	0.085

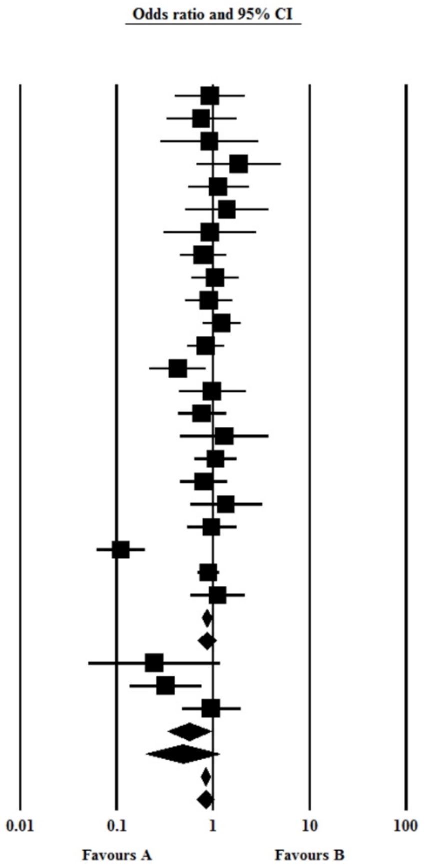

Fig. S6. Forest plot of the association between B blood group and *H. pylori* infection according to NOS quality scale. For each study, the box represents the random effects odds ratio and the line the 95% confidence intervals. The size of each box indicates the relative weight of each study in the meta-analysis. Test for overall fixed effects: High, z = -2.627; P = 0.009; Low, z = -2.198; P = 0.028; Overall, z = -3.063; P = 0.085.

Model	Group by Study Design	Study name	Odds ratio	Lower limit	Upper limit	Z-Value	p-Value
	Cohort	Robertson, 2003	1.040	0.594	1.820	0.137	0.891
	Cohort	Bhuiyan, 2009	0.901	0.511	1.586	-0.362	0.717
Fixed	Cohort		0.969	0.651	1.442	-0.157	0.875
Random	Cohort		0.969	0.651	1.442	-0.157	0.875
	Cross-sectional	Niv, 1996	0.926	0.400	2.144	-0.180	0.857
	Cross-sectional	Valliani, 2013	0.246	0.051	1.183	-1.750	0.080
	Cross-sectional	Umlauft, 1996	0.754	0.325	1.746	-0.659	0.510
	Cross-sectional	Rasmi,2011	0.320	0.135	0.762	-2.576	0.010
	Cross-sectional	Petrovic, 2011	0.952	0.475	1.911	-0.137	0.891
	Cross-sectional	Klaamas, 1997	0.917	0.284	2.956	-0.146	0.884
	Cross-sectional	Yei, 2005	1.854	0.679	5.065	1.204	0.229
	Cross-sectional	Loffeld, 1991	1.133	0.549	2.338	0.339	0.735
	Cross-sectional	Heneghan, 1998	1.389	0.513	3.762	0.646	0.518
	Cross-sectional	deMattos, 2002	0.923	0.306	2.788	-0.142	0.887
	Cross-sectional	Turkolmez, 2007	0.783	0.449	1.367	-0.860	0.390
	Cross-sectional	Jafarzadeh, 2007	1.233	0.783	1.943	0.905	0.365
	Cross-sectional	Kanbay, 2005	0.836	0.537	1.300	-0.795	0.427
	Cross-sectional	Ansari, 2015	0.429	0.218	0.844	-2.450	0.014
	Cross-sectional	Keller, 2002	0.978	0.443	2.160	-0.055	0.956
	Cross-sectional	Bayan, 2008	0.766	0.434	1.353	-0.918	0.359
	Cross-sectional	Oberhuber, 1997	1.308	0.455	3.755	0.499	0.618
	Cross-sectional	Wu, 2003	1.059	0.641	1.750	0.223	0.823
	Cross-sectional	Tadesse, 2014	0.794	0.449	1.401	-0.797	0.425
	Cross-sectional	Moges, 2006	1.357	0.573	3.213	0.694	0.488
	Cross-sectional	Klaamas, 1999	0.964	0.533	1.743	-0.122	0.903
	Cross-sectional	Lin, 1998	0.110	0.061	0.198	-7.390	0.000
	Cross-sectional	Nakao, 2011	0.890	0.691	1.147	-0.902	0.367
	Cross-sectional	Hook-Nikanne,1990	1.108	0.575	2.138	0.307	0.759
Fixed	Cross-sectional		0.819	0.723	0.927	-3.159	0.002
Random	Cross-sectional		0.806	0.639	1.018	-1.809	0.070
Fixed	Overall		0.831	0.738	0.936	-3.063	0.002
Random	Overall		0.845	0.691	1.033	-1.640	0.101

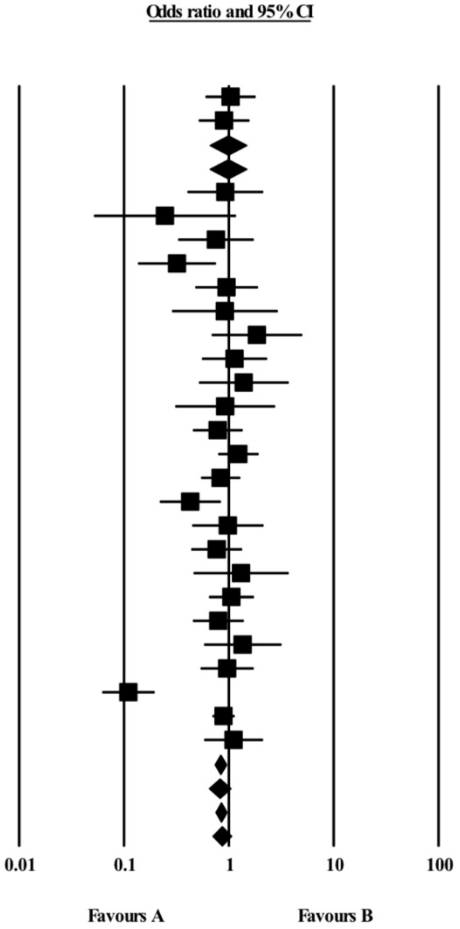

Fig. S7. Forest plot of the association between B blood group and *H. pylori* infection according to NOS quality scale. For each study, the box represents the random effects odds ratio and the line the 95% confidence intervals. The size of each box indicates the relative weight of each study in the meta-analysis. Test for overall fixed effects: High, z = -2.627; P = 0.009; Low, z = -2.198; P = 0.028; Overall, z = -3.063; P = 0.085.

Study name	Statistics with study removed				
	Point	Lower limit	Upper limit	Z-Value	p-Value
Niv, 1996	0.829	0.736	0.934	-3.078	0.002
Valliani, 2013	0.837	0.743	0.942	-2.950	0.003
Umlauft, 1996	0.832	0.739	0.938	-3.010	0.003
Rasmi,2011	0.846	0.751	0.953	-2.747	0.006
Petrovic, 2011	0.827	0.734	0.933	-3.095	0.002
Klaamas, 1997	0.830	0.737	0.935	-3.074	0.002
Yei, 2005	0.822	0.729	0.925	-3.237	0.001
Loffeld, 1991	0.824	0.731	0.929	-3.171	0.002
Heneghan, 1998	0.825	0.732	0.929	-3.172	0.002
deMattos, 2002	0.830	0.737	0.935	-3.076	0.002
Turkolmez, 2007	0.823	0.730	0.928	-3.180	0.001
Robertson, 2003	0.822	0.728	0.928	-3.174	0.002
Bhuiyan, 2009	0.828	0.734	0.934	-3.065	0.002
Henrikkson, 1993	0.831	0.738	0.936	-3.063	0.002
Jafarzadeh, 2007	0.807	0.714	0.912	-3.426	0.001
Kanbay, 2005	0.830	0.735	0.939	-2.969	0.003
Ansari, 2015	0.848	0.752	0.956	-2.687	0.007
Keller, 2002	0.828	0.734	0.933	-3.100	0.002
Bayan, 2008	0.834	0.739	0.941	-2.947	0.003
Oberhuber, 1997	0.826	0.733	0.930	-3.149	0.002
Wu, 2003	0.819	0.725	0.925	-3.216	0.001
Tadesse, 2014	0.833	0.738	0.939	-2.972	0.003
Moges, 2006	0.823	0.730	0.927	-3.198	0.001
Klaamas, 1999	0.826	0.732	0.932	-3.111	0.002
Lin, 1998	0.905	0.802	1.022	-1.613	0.107
Nakao, 2011	0.815	0.713	0.932	-2.998	0.003
Hook-Nikanne,1990	0.823	0.730	0.928	-3.180	0.001
	0.831	0.738	0.935	-3.073	0.002

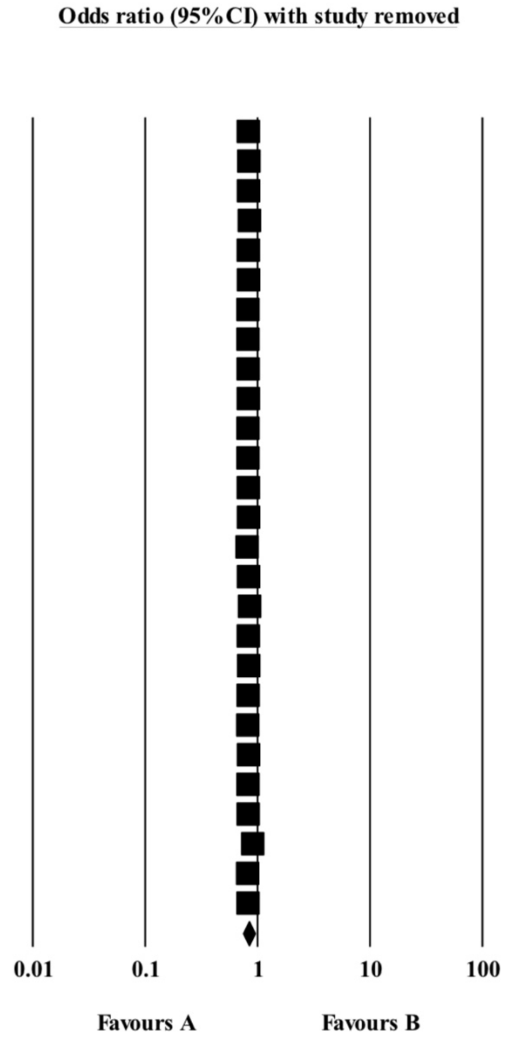

Fig. S8. Sensitivity analysis for B blood group and *H. pylori*. The vertical axis shows the omitted study. The box represents the random effects odds ratio and the line the 95% confidence intervals when the named study is omitted from the meta-analysis.

Model	Group by Development	Study name	Odds ratio	Lower limit	Upper limit	Z-Value	p-Value
	Developed	Umlauft, 1996	1.099	0.271	4.466	0.132	0.895
	Developed	Klaamas, 1997	0.902	0.240	3.391	-0.153	0.879
	Developed	Heneghan, 1998	0.591	0.106	3.303	-0.599	0.549
	Developed	Robertson, 2003	1.678	0.613	4.588	1.008	0.314
	Developed	Henrikkson, 1993	1.949	0.087	43.784	0.420	0.674
	Developed	Keller, 2002	1.465	0.528	4.059	0.734	0.463
	Developed	Oberhuber, 1997	0.634	0.177	2.266	-0.701	0.483
	Developed	Klaamas, 1999	1.771	0.609	5.154	1.049	0.294
	Developed	Nakao, 2011	0.832	0.581	1.190	-1.008	0.313
	Developed	Hook-Nikanne,1990	0.755	0.321	1.776	-0.643	0.520
Fixed	Developed		0.942	0.722	1.229	-0.439	0.661
Random	Developed		0.942	0.722	1.229	-0.439	0.661
	Developing	Niv, 1996	6.811	0.865	53.613	1.822	0.068
	Developing	Valliani, 2013	0.514	0.051	5.144	-0.566	0.571
	Developing	Rasmi,2011	1.399	0.703	2.786	0.956	0.339
	Developing	Petrovic, 2011	0.391	0.139	1.101	-1.778	0.075
	Developing	Yei, 2005	0.656	0.133	3.235	-0.518	0.605
	Developing	deMattos, 2002	0.930	0.149	5.785	-0.078	0.938
	Developing	Turkolmez, 2007	1.014	0.536	1.916	0.041	0.967
	Developing	Bhuiyan, 2009	0.578	0.254	1.311	-1.312	0.189
	Developing	Jafarzadeh, 2007	0.907	0.455	1.809	-0.276	0.782
	Developing	Kanbay, 2005	0.149	0.080	0.278	-5.994	0.000
	Developing	Ansari, 2015	0.505	0.236	1.079	-1.763	0.078
	Developing	Bayan, 2008	0.491	0.194	1.247	-1.495	0.135
	Developing	Wu, 2003	0.999	0.404	2.474	-0.001	0.999
	Developing	Tadesse, 2014	1.630	0.366	7.257	0.641	0.522
	Developing	Moges, 2006	0.659	0.133	3.260	-0.511	0.609
	Developing	Lin, 1998	0.511	0.350	0.745	-3.488	0.000
Fixed	Developing		0.601	0.492	0.735	-4.963	0.000
Random	Developing		0.669	0.463	0.967	-2.139	0.032
Fixed	Overall		0.708	0.603	0.831	-4.224	0.000
Random	Overall		0.838	0.675	1.040	-1.607	0.108

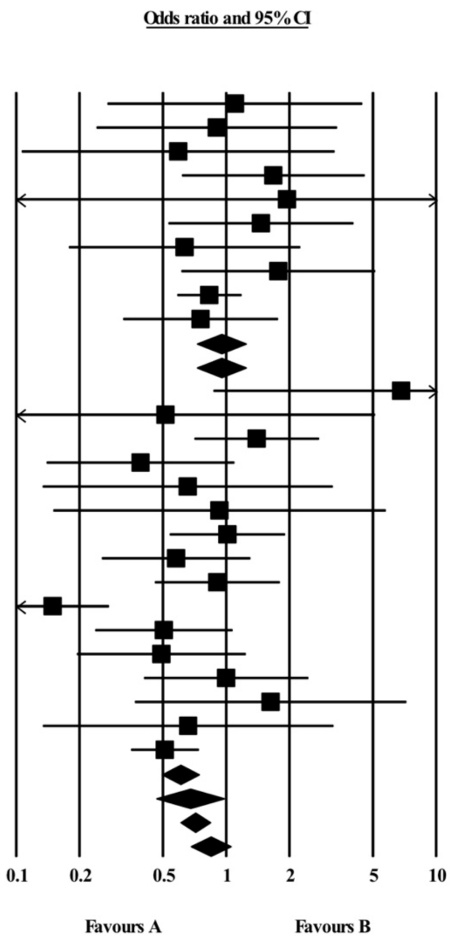

Fig. S9. Forest plot of the association between AB blood group and H. pylori infection according to definition of income. Developed indicates studies completed in high income countries and developing indicates studies completed in low income countries. For each study, the box represents the random effects odds ratio and the line the 95% confidence intervals. The size of each box indicates the relative weight of each study in the meta-analysis. Test for overall fixed effects: Developed; z = -0.439, P = 0.661 Developing, z = -4.963; P < 0.001; Overall z = 4.224; P < 0.001.

Model	Group by Study Quality	Study name	Odds ratio	Lower limit	Upper limit	Z-Value	p-Value
	High	Niv, 1996	6.811	0.865	53.613	1.822	0.068
	High	Umlauft, 1996	1.099	0.271	4.466	0.132	0.895
	High	Klaamas, 1997	0.902	0.240	3.391	-0.153	0.879
	High	Yei, 2005	0.656	0.133	3.235	-0.518	0.605
	High	Loffeld, 1991	0.799	0.242	2.643	-0.367	0.714
	High	Heneghan, 1998	0.591	0.106	3.303	-0.599	0.549
	High	deMattos, 2002	0.930	0.149	5.785	-0.078	0.938
	High	Turkolmez, 2007	1.014	0.536	1.916	0.041	0.967
	High	Robertson, 2003	1.678	0.613	4.588	1.008	0.314
	High	Bhuiyan, 2009	0.578	0.254	1.311	-1.312	0.189
	High	Jafarzadeh, 2007	0.907	0.455	1.809	-0.276	0.782
	High	Kanbay, 2005	0.149	0.080	0.278	-5.994	0.000
	High	Ansari, 2015	0.505	0.236	1.079	-1.763	0.078
	High	Keller, 2002	1.465	0.528	4.059	0.734	0.463
	High	Bayan, 2008	0.491	0.194	1.247	-1.495	0.135
	High	Oberhuber, 1997	0.634	0.177	2.266	-0.701	0.483
	High	Wu, 2003	0.999	0.404	2.474	-0.001	0.999
	High	Tadesse, 2014	1.630	0.366	7.257	0.641	0.522
	High	Moges, 2006	0.659	0.133	3.260	-0.511	0.609
	High	Klaamas, 1999	1.771	0.609	5.154	1.049	0.294
	High	Lin, 1998	0.511	0.350	0.745	-3.488	0.000
	High	Nakao, 2011	0.832	0.581	1.190	-1.008	0.313
	High	Hook-Nikanne,1990	0.755	0.321	1.776	-0.643	0.520
Fixed	High		0.692	0.586	0.816	-4.353	0.000
Random	High		0.760	0.578	1.000	-1.961	0.050
	Low	Valliani, 2013	0.514	0.051	5.144	-0.566	0.571
	Low	Rasmi,2011	1.399	0.703	2.786	0.956	0.339
	Low	Petrovic, 2011	0.391	0.139	1.101	-1.778	0.075
Fixed	Low		0.913	0.523	1.592	-0.321	0.748
Random	Low		0.757	0.287	1.997	-0.563	0.573
Fixed	Overall		0.707	0.603	0.829	-4.262	0.000
Random	Overall		0.760	0.584	0.989	-2.040	0.041

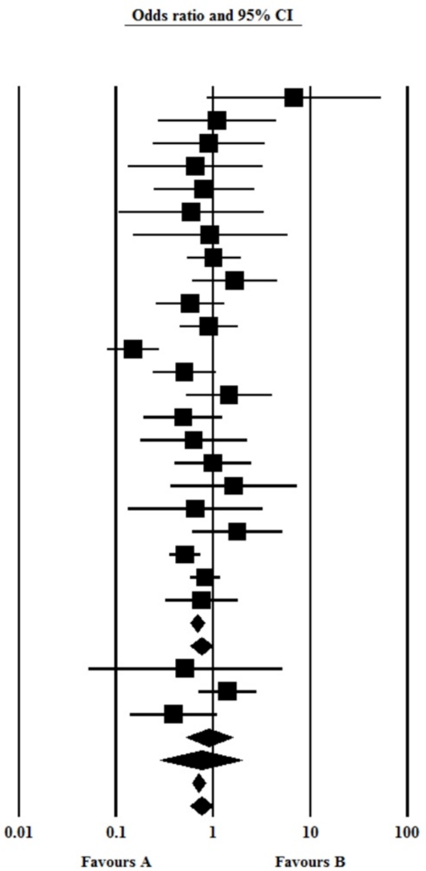

Fig. S10. Forest plot of the association between AB blood group and *H. pylori* infection according to NOS quality scale. For each study, the box represents the random effects odds ratio and the line the 95% confidence intervals. The size of each box indicates the relative weight of each study in the meta-analysis. Test for overall fixed effects: High, z = -4.353; P < 0.001; Low, z = -0.321; P = 0.748; Overall, z = -4.262; P < 0.001.

Model	Group by Study design	Study name	Odds ratio	Lower limit	Upper limit	Z-Value	p-Value
	Cohort	Robertson, 2003	1.678	0.613	4.588	1.008	0.314
	Cohort	Bhuiyan, 2009	0.578	0.254	1.311	-1.312	0.189
Fixed	Cohort		0.884	0.468	1.669	-0.381	0.703
Random	Cohort		0.944	0.333	2.676	-0.108	0.914
	Cross-sectional	Niv, 1996	6.811	0.865	53.613	1.822	0.068
	Cross-sectional	Valliani, 2013	0.514	0.051	5.144	-0.566	0.571
	Cross-sectional	Umlauft, 1996	1.099	0.271	4.466	0.132	0.895
	Cross-sectional	Rasmi,2011	1.399	0.703	2.786	0.956	0.339
	Cross-sectional	Petrovic, 2011	0.391	0.139	1.101	-1.778	0.075
	Cross-sectional	Klaamas, 1997	0.902	0.240	3.391	-0.153	0.879
	Cross-sectional	Yei, 2005	0.656	0.133	3.235	-0.518	0.605
	Cross-sectional	Loffeld, 1991	0.799	0.242	2.643	-0.367	0.714
	Cross-sectional	Heneghan, 1998	0.591	0.106	3.303	-0.599	0.549
	Cross-sectional	deMattos, 2002	0.930	0.149	5.785	-0.078	0.938
	Cross-sectional	Turkolmez, 2007	1.014	0.536	1.916	0.041	0.967
	Cross-sectional	Jafarzadeh, 2007	0.907	0.455	1.809	-0.276	0.782
	Cross-sectional	Kanbay, 2005	0.149	0.080	0.278	-5.994	0.000
	Cross-sectional	Ansari, 2015	0.505	0.236	1.079	-1.763	0.078
	Cross-sectional	Keller, 2002	1.465	0.528	4.059	0.734	0.463
	Cross-sectional	Bayan, 2008	0.491	0.194	1.247	-1.495	0.135
	Cross-sectional	Oberhuber, 1997	0.634	0.177	2.266	-0.701	0.483
	Cross-sectional	Wu, 2003	0.999	0.404	2.474	-0.001	0.999
	Cross-sectional	Tadesse, 2014	1.630	0.366	7.257	0.641	0.522
	Cross-sectional	Moges, 2006	0.659	0.133	3.260	-0.511	0.609
	Cross-sectional	Klaamas, 1999	1.771	0.609	5.154	1.049	0.294
	Cross-sectional	Lin, 1998	0.511	0.350	0.745	-3.488	0.000
	Cross-sectional	Nakao, 2011	0.832	0.581	1.190	-1.008	0.313
	Cross-sectional	Hook-Nikanne,1990	0.755	0.321	1.776	-0.643	0.520
Fixed	Cross-sectional		0.697	0.591	0.822	-4.304	0.000
Random	Cross-sectional		0.751	0.571	0.986	-2.063	0.039
Fixed	Overall		0.707	0.603	0.829	-4.262	0.000
Random	Overall		0.762	0.585	0.992	-2.023	0.043

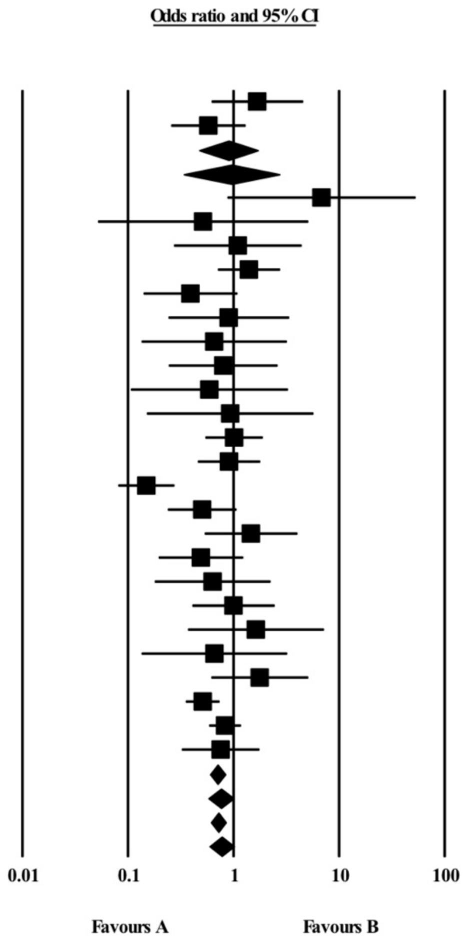

Fig. S11 Forest plot of the association between AB blood group and H. pylori infection according to study design. For each study, the box represents the random effects odds ratio and the line the 95% confidence intervals. The size of each box indicates the relative weight of each study in the meta-analysis. Test for overall fixed effects: Cohort, z = -0.381; P = 0.703; Cross-sectional, z = -4.304; P < 0.001; Overall, z = -4.262; P < 0.001.

Study name	Statistics with study removed				
	Point	Lower limit	Upper limit	Z-Value	p-Value
Niv, 1996	0.700	0.597	0.821	-4.389	0.000
Valliani, 2013	0.710	0.606	0.833	-4.206	0.000
Umlauft, 1996	0.705	0.601	0.828	-4.278	0.000
Rasmi,2011	0.683	0.580	0.804	-4.580	0.000
Petrovic, 2011	0.720	0.613	0.845	-4.010	0.000
Klaamas, 1997	0.707	0.602	0.830	-4.248	0.000
Yei, 2005	0.710	0.605	0.833	-4.205	0.000
Loffeld, 1991	0.708	0.603	0.831	-4.224	0.000
Heneghan, 1998	0.710	0.606	0.833	-4.198	0.000
deMattos, 2002	0.708	0.603	0.830	-4.245	0.000
Turkolmez, 2007	0.693	0.588	0.816	-4.385	0.000
Robertson, 2003	0.694	0.591	0.815	-4.451	0.000
Bhuiyan, 2009	0.715	0.608	0.841	-4.058	0.000
Henrikkson, 1993	0.707	0.603	0.829	-4.262	0.000
Jafarzadeh, 2007	0.700	0.594	0.824	-4.287	0.000
Kanbay, 2005	0.791	0.671	0.932	-2.797	0.005
Ansari, 2015	0.720	0.612	0.848	-3.954	0.000
Keller, 2002	0.697	0.593	0.818	-4.404	0.000
Bayan, 2008	0.717	0.610	0.843	-4.040	0.000
Oberhuber, 1997	0.711	0.605	0.834	-4.181	0.000
Wu, 2003	0.702	0.597	0.825	-4.302	0.000
Tadesse, 2014	0.703	0.599	0.824	-4.328	0.000
Moges, 2006	0.710	0.605	0.833	-4.205	0.000
Klaamas, 1999	0.695	0.592	0.816	-4.441	0.000
Lin, 1998	0.761	0.639	0.907	-3.051	0.002
Nakao, 2011	0.682	0.571	0.814	-4.227	0.000
Hook-Nikanne,1990	0.708	0.602	0.832	-4.189	0.000
	0.709	0.605	0.832	-4.235	0.000

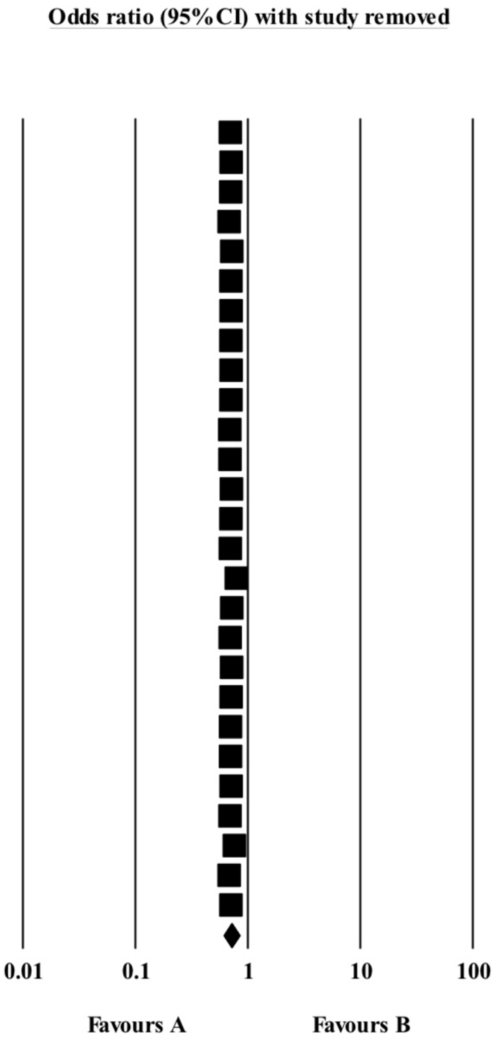

Fig. S12. Sensitivity analysis for AB blood group and *H. pylori*. The vertical axis shows the omitted study. The box represents the random effects odds ratio and the line the 95% confidence intervals when the named study is omitted from the meta-analysis.

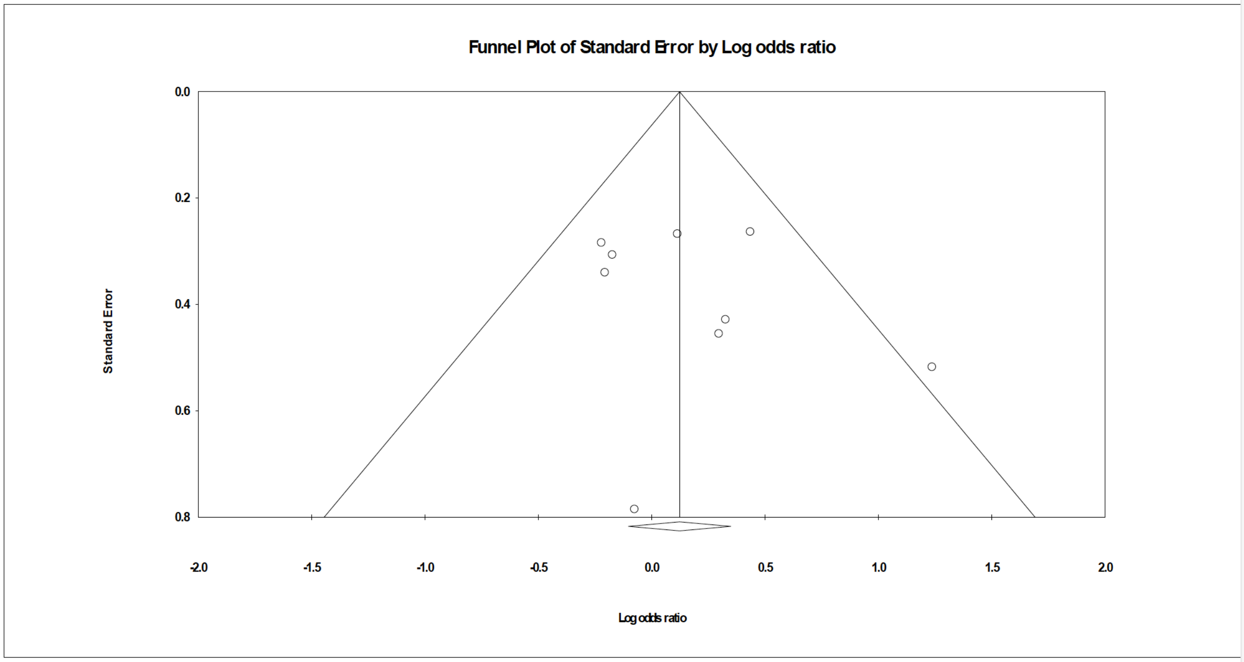

Fig. S13. Funnel plot of standard error by log odds ration for the association between secretor status and *H. pylori* infection. Egger's test; b = 1.0672, P = 0.441
